# Sex-Dependent Dynamics of Behavioural and Neuropathological Changes in an A53T Alpha-Synuclein Mouse Model of Parkinson’s Disease

**DOI:** 10.1007/s10571-026-01707-9

**Published:** 2026-03-04

**Authors:** Maider Zubelzu, Raphaelle Bidgood, Ane Murueta-Goyena, José Ángel Ruiz-Ortega, José Vicente Lafuente, Teresa Morera-Herreras

**Affiliations:** 1 LaNCE-Neuropharm Research Group, Department of Pharmacology, Faculty of Medicine and Nursery, University of the Basque Country (EHU), Leioa, Spain; 2 Neurodegenerative Diseases Group, Biobizkaia Health Research Institute, Barakaldo, Bizkaia Spain; 3 LaNCE-Neuropharm Research Group, Department of Neurosciences, Faculty of Medicine and Nursery, University of the Basque Country (EHU), Leioa, Spain

**Keywords:** Parkinson’s disease, Sex differences, A53T alpha-synuclein, Motor impairment, Axonal degeneration, Neuroinflammation

## Abstract

Parkinson’s disease (PD) is characterised by progressive dopaminergic neurodegeneration, alpha-synuclein (α-syn) aggregation, and neuroinflammation. Clinical and experimental studies suggest that sex influences disease onset, progression, and treatment response, yet its impact on α-syn-mediated pathology remains incompletely understood. In this study, we investigated sex-dependent progression of behavioural and neuropathological alterations in a mouse model overexpressing A53T α-syn. Male and female C57BL/6J mice received bilateral intranigral injections of adeno-associated viral vectors encoding mutant A53T α-syn or empty vectors. Motor performance was assessed at 60 and 120 days post-surgery using open field, wire hang, pole, and balance beam tasks complemented by automated behavioural analysis (DeepLabCut, SimBA). Brains were processed for immunohistochemical evaluation of α-syn accumulation, tyrosine hydroxylase (TH) expression, axonal integrity, and glial activation. A53T α-syn overexpression induced early subtle motor deficits primarily in males, accompanied by increased immobility on the balance beam despite preserved substantia nigra compacta neuron counts. By 120 days, striatal TH immunoreactivity was markedly reduced in α-syn mice. An integrated axonal degeneration index, combining TH optical density and swelling counts, revealed a faster progression of striatal axonopathy in males. Both sexes showed enhanced striatal astrogliosis, indicating α-syn-driven neuroinflammation independent of sex. These findings identify early axonal degeneration and reactive astrogliosis as key pathological events preceding neuronal loss in A53T α-syn mice. Importantly, the pronounced male vulnerability highlights sex as a critical biological variable in modelling PD progression and in developing precision-based neuroprotective strategies.

**Graphical Abstract:**

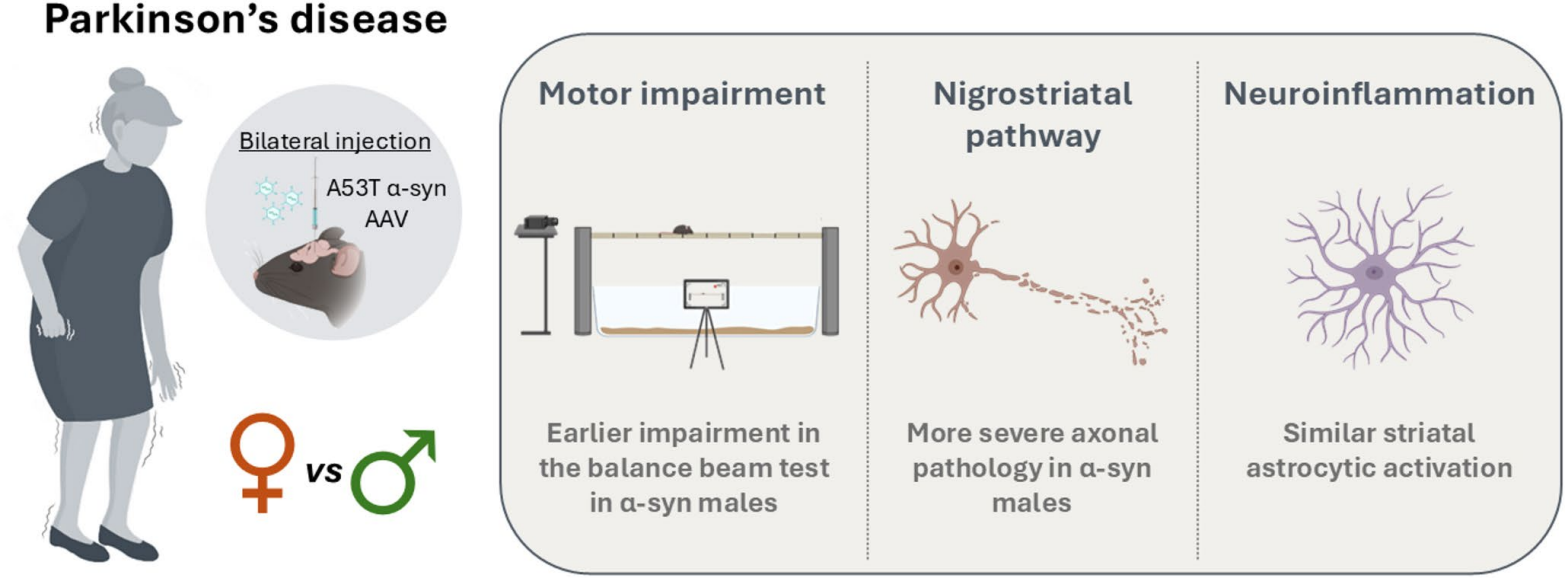

**Supplementary Information:**

The online version contains supplementary material available at 10.1007/s10571-026-01707-9.

## Introduction

Parkinson’s disease (PD) is a progressive neurodegenerative disorder characterised primarily by motor dysfunction, including bradykinesia, rigidity, resting tremor, and postural instability, as well as a range of non-motor symptoms (Mirelman et al. 2019; Tysnes and Storstein 2017). The pathological hallmark of PD is the gradual loss of dopaminergic neurons in the *substantia nigra pars compacta* (SNc), resulting in striatal dopamine depletion. In parallel, PD is marked by the accumulation of misfolded alpha-synuclein (α-syn) aggregates in the form of Lewy bodies and Lewy neurites, which are considered central to disease pathogenesis (Blesa et al. 2022; Choong and Mochizuki 2022; Sharma and Burré 2023).

Observational clinical studies have consistently reported sex differences in PD incidence, progression, and symptomatology. Men are more likely to develop PD than women (Weber and Clyne 2021), whereas women often experience a higher burden of non-motor symptoms such as anxiety, depression, and dysautonomia (Zachry et al. 2021). The biological underpinnings of these sex differences remain poorly understood but are thought to involve complex interactions between sex hormones, genetic factors, and neuroinflammatory processes (Rugbjerg et al. 2013; Zachry et al. 2021).

α-Syn plays a central role in PD pathogenesis, both as a principal component of Lewy pathology and as a driver of neuronal dysfunction and degeneration. Overexpression of mutant forms of α-syn, such as the A53T variant linked to familial PD, recapitulates key pathological features of the disease (Sharma and Burré 2023). Viral vector-mediated α-syn overexpression using adeno-associated viruses (AAV) has become a widely used approach to model PD in rodents, enabling targeted, controlled expression of α-syn in specific brain regions (Koprich et al. 2017). These models reproduce dopaminergic neurodegeneration, motor deficits, synucleinopathy, and neuroinflammation, providing valuable platforms for studying disease mechanisms and testing therapeutic interventions.

Despite increasing use of AAV-mediated α-syn overexpression models, most studies have not systematically examined potential sex differences in α-syn-driven pathology and behavioural outcomes. This represents a significant gap in current knowledge, particularly in light of the sex differences observed in human PD. Understanding how sex modulates vulnerability to α-syn-induced neurodegeneration could provide critical insights into disease mechanisms and support the development of more effective, personalised therapeutic strategies.

In the present study, we investigated whether AAV-mediated overexpression of A53T α-syn induces sex-specific differences in behavioural, neurochemical, and neuropathological outcomes in mice. To this end, we systematically assessed motor behaviour, dopaminergic neurodegeneration, and neuroinflammatory responses over time in both male and female animals, to better understand if sex influences the progression of α-syn-mediated pathology.

## Materials and Methods

### Animals and Ethical Statement

7-week-old male and female C57BL/6J mice (Janvier Labs) were housed in groups of 3–6 in individually ventilated cages under standard laboratory conditions (22 ± 1 °C, 55 ± 5% relative humidity, and a 12:12 h light/dark cycle, with the light phase from 8am to 8pm) with food and water provided *ad libitum*. Every effort was made to minimise animal suffering and to use the minimum number of animals per group and experiment. The experimental protocol was reviewed and approved by the Local Ethical Committee for Animal Research of the University of the Basque Country (EHU, CEEA, protocols M20/2019/219, M20-2022-215 and M30/2019/220). All experiments were performed in accordance with the European Community Council Directive on “The Protection of Animals Used for Scientific Purposes” (2010/63/EU) and with the Spanish law (RD 53/2013) on the care and use of laboratory animals.

### Experimental Design

Sample size determination was based on both behavioural and histological endpoints. Published studies of AAV-mediated α-syn overexpression in mice report large effect sizes at symptomatic stages (Cohen’s d ≈ 1.5–2.0) (Hong et al. 2025; Oliveras-Salvá et al. 2013; Song et al. 2015). Based on these data and assuming a two-way ANOVA (treatment × sex) with a two-sided α of 0.05 and 80% power, power calculations indicated that approximately 8 animals per group at the final time point would be sufficient to detect a main effect of treatment, using a conservative effect size of d = 1.5. Larger cohorts (10–15 animals per group) were therefore initially enrolled to accommodate interim sacrifice at 60 days and potential attrition. For tyrosine hydroxylase immunostaining, 5 animals per group were used, consistent with prior AAV-α-syn studies reporting large effect sizes and low variability for dopaminergic neuron measures (Gombash et al. 2013; Landeck et al. 2017).

This study used a total of 28 male (21–25 g) and 27 female (16–22 g) C57BL/6J mice. AAV vectors encoding for mutant A53T human α-syn or empty vectors were injected bilaterally into the SNc. One female mouse that had been injected with the mutant A53T α-syn was excluded due to the incorrect viral diffusion in one hemisphere. Animals were weighed every week to monitor possible abnormal weight changes.

To investigate the behavioural and morphological alterations in neurons and glial cells within the nigrostriatal pathway, the animals were sacrificed at 60 or 120 days after the stereotaxic injection.

Two batches of animals were used in this study: 16 mice in batch 1 and 40 mice in batch 2. Animals of batch 1 underwent behavioural testing at 60 days post-surgery before being sacrificed at this time point, while the mice of batch 2 performed the behavioural tests at 60 and 120 days post-surgery and were all sacrificed at 120 days post-surgery. Immunohistochemistry was carried out independently at both time points (at 60 days post-surgery for batch 1 and at 120 days for batch 2). Animals with insufficient viral transduction were excluded prior to data analysis, based on pre-established histological criteria. Insufficient transduction was defined as a lack of detectable human A53T α-syn immunoreactivity in the SNc and/or the absence of α-syn-positive fibres in the ipsilateral striatum, as assessed post hoc by immunohistochemistry. The sample sizes for each study are detailed in Table 1.

**Table 1:** Summary of the sample sizes used in the behavioural and morphological studies

Experimental group	n	
	Behavioural study	Morphological study
**60 days**		
*Male*		
Empty vector	13	3
A53T α-syn	15	5
*Female*		
Empty vector	13	3
A53T α-syn	14	5
**120 days**		
*Male*		
Empty vector	10	5
A53T α-syn	10	5
*Female*		
Empty vector	10	5
A53T α-syn	9	4

Both studies were conducted at 60 and 120 days post-surgery on male and female mice that had been injected with adeno-associated viral (AAV) vectors encoding either A53T mutant human alpha-synuclein (α-syn) or the empty vector. Mice sacrificed at 120 days post-surgery performed the behavioural tests at 60 and 120 days post-surgery, and some of the brains were used for the morphological study

To minimise subjective bias, mice were randomly assigned to experimental groups (AAV-A53T α-syn versus empty vector) immediately after surgery and were stratified by sex and body weight. Littermates were distributed across the experimental groups to control for potential litter effects. Experimenters were not blinded to group allocation during behavioural testing, tissue processing, immunohistochemical quantification, and all statistical analyses.

### AAV Surgical Procedure

AAV vectors (rAAV9-CMVie/SynP-WPRE) encoding either mutant A53T human α-syn (1 × 10^13^ genomic particles/ml) or the empty vector (3.1 × 10^12^ genomic particles/ml) were obtained from the University of Bordeaux (France). The AAV-A53T α-syn vector was used diluted in Lactated Ringer’s Solution at the supplier’s recommended titre for reliable SNc transduction, while the empty vector was employed undiluted at its provided native titre (rather than further diluting the AAV-A53T preparation to match titres). Mice were anaesthetised with isoflurane (induction 5%; maintenance 1.5–2%) in oxygen-enriched air (1–2%) and placed in a stereotaxic frame (David Kopf^®^ Instruments). Body temperature was maintained at ~ 37 °C throughout the experiment using a heating pad connected to a rectal probe. AAV vectors were then bilaterally injected into the SNc (1 µl per hemisphere) using a glass pipette (coordinates from Bregma: anteroposterior (AP) − 2.9 mm; mediolateral (ML) ± 1.4 mm and dorsoventral (DV) − 4.5 mm). The pipette was left in place for 5 min after the injection to prevent leakage. Meloxicam (2 mg/kg) was administered subcutaneously to relieve pain and/or reduce inflammation.

### Behavioural Assessment

**Fig. 1:**
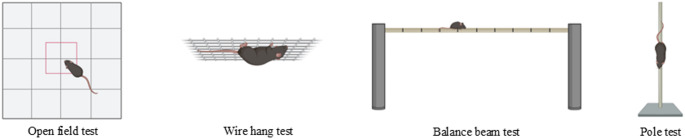
Illustrative images of the behavioural tests conducted in this study. The open field, wire hang, balance beam and pole tests were all performed at both 60 and 120 days post-surgery. Adapted from BioRender and not drawn to scale

Several behavioural tests were performed at 60 and 120 days post-surgery to investigate the possible motor changes induced by the overexpression of mutant A53T human α-syn (Fig. 1).

### Open Field Test

The open field test is a common measure of spontaneous motor activity in mice. In this test, a mouse was placed in the centre of a square arena, and its movements were automatically recorded using Actitrack software (Panlab, Spain). The square arena (44 × 44 × 35 cm) uses parallel infrared beams spaced 2.5 cm apart to track the animal’s motion accurately. Mice were placed in the centre of the arena and were given 10 min to explore (acclimatisation), followed by 10 min of data collection. After each session, the arena was cleaned with 70° alcohol and the sawdust was replaced before another animal was placed into the arena. The system measured various parameters, including total activity, total distance travelled, resting time, vertical activity, speed and time spent performing fast (more than 5 cm/s), slow (less than 5 cm/s) and stereotyped movements. The automated tracking also distinguished between central and peripheral zones of the arena, allowing the analysis of the time the mouse spent in each area.

### Wire Hang Test

The wire hang test (grid test) was used to evaluate motor coordination and muscle strength in mice. Each mouse was placed onto a standard cage grid, which was gently shaken to encourage gripping, and then turned upside down approximately 50 cm above a sawdust-filled cage. The time it took for each mouse to fall was recorded, with a maximum test duration of 15 min. The test was conducted on two consecutive days.

### Balance Beam Test

**Fig. 2:**
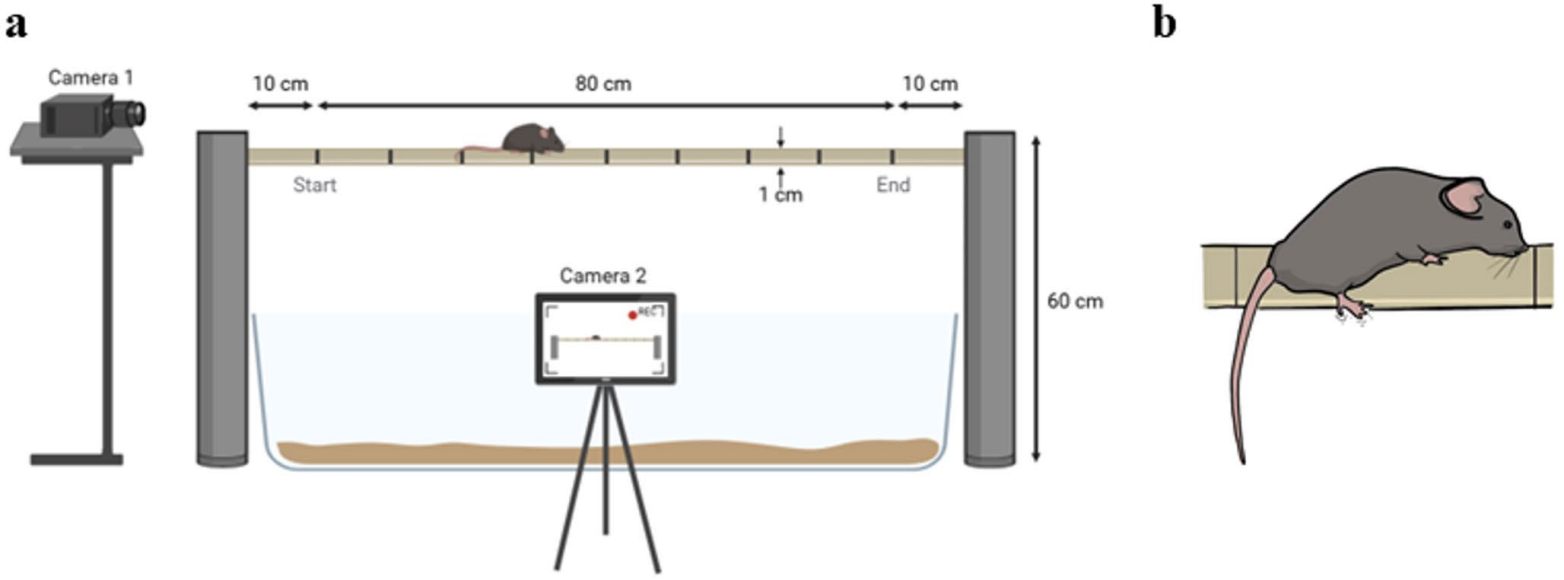
Illustrative images of the balance beam test. **a** Schematic representation of the experimental setup for the balance beam test, which assesses the ability of the mouse to walk across a wooden beam (80 cm long and 1 cm wide at a height of 60 cm). Cameras 1 and 2 (placed at the rear and laterally to the beam, respectively) recorded each trial for in-depth manual and automated analyses, post-behavioural sessions. **b** Illustration of a hind paw slip, defined as the back foot leaving the top of the beam. Adapted from BioRender and not drawn to scale (Bidgood et al. 2024)

The beam-walking test is used in rodents to measure subtle motor and balance impairments that may not be detected by other tests like the rotarod. Mice were trained and then tested on their ability to walk across a narrow (1 cm wide), elevated (60 cm high) 80 cm-long wooden beam (Fig. 2a). The mouse was placed in front of the start line and was removed from the beam either when it had crossed the finish line or when the time on the beam exceeded 1 min. Each mouse performed three trials, separated by 10 min, and mouse performances were then averaged. Prior to the first trial, mice were acclimatised to the behavioural room for 30 min. One day prior to testing, mice were trained to cross the beam under the same conditions as the test (3 trials per animal, with a 10-minute interval between each trial). Test trials were recorded using two cameras (Fig. 2a): one for manual quantification of hind paw slips (Fig. 2b) and another for automated tracking of the animal to analyse the mouse walking pattern along the beam. A slip was defined as the foot leaving the top of the beam.

The automated analysis followed the pipeline previously published by Bidgood et al. (2024), using the open-source tools DeepLabCut (version 2.2.3; Nath et al. 2019) and Simple Behavioral Analysis (SimBA; version 2.8.5; Goodwin et al. 2024). Briefly, DeepLabCut enabled the automated tracking of the animal along the beam using markerless pose estimation (with 5 keypoints: “Nose”, “Head”, “Bodytop”, “Bodymiddle” and “Tailbase”), while SimBA facilitated the automated behavioural classification to detect mouse walking patterns. Specifically, it was used to measure the percentage of walking (i.e. classifier probability of occurrence), the overall time spent walking (i.e. total event duration) and to quantify walking bouts. Each bout was defined as a sequence of uninterrupted walking. These bouts were quantified based on metrics, such as the number of bouts (i.e. uninterrupted walking episodes), the mean and median walking bout duration, and mean and median bout interval duration (i.e. the time during which the animal has ceased walking and remains immobile and/or frozen). The DeepLabCut/SimBA pipeline also enabled the automated measurement of the time to cross the beam, and the maximum distance travelled using regions of interest (ROIs). The beam was divided into 10 ROIs, each 10 cm long. SimBA provided the number of entries in each ROI as well as the time spent in each one (considering the “Bodymiddle” body-part as the reference). Animals entering a ROI at least once were considered to have crossed the corresponding line, aided to calculate the maximum distance covered. Values of the times spent in each ROI of the beam were summed to determine the time to cross the beam. If a mouse never crossed the start line, the trial was excluded from the manual and automated analyses and given the default values of a failed trial (60 s and 0 cm).

### Pole Test

**Fig. 3:**
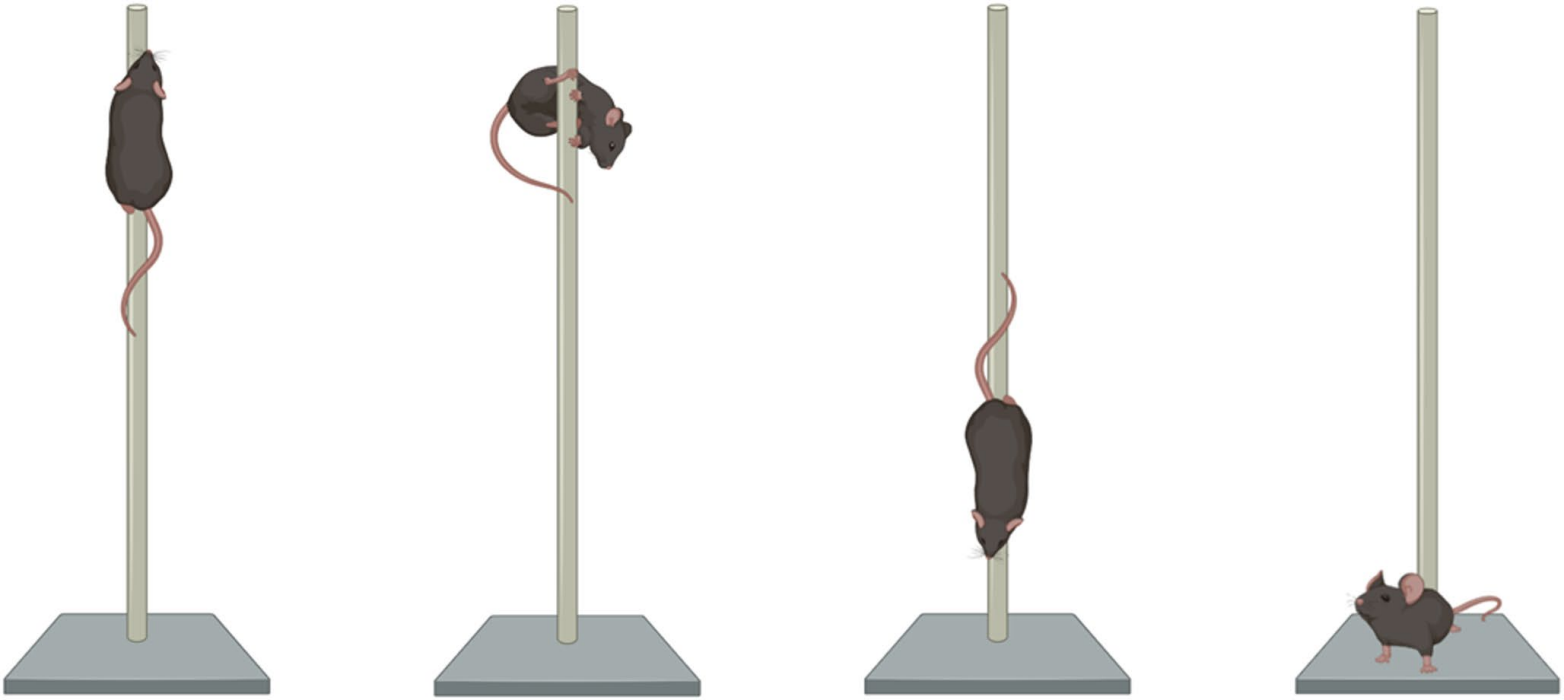
Illustrative images of the pole test. The mouse is first placed on the top of the pole, facing upwards, marking the start of the test. Using a manual chronometer, the time the mouse takes to turn downwards is measured, as well as the subsequent descent time and the total time spent on the pole. The test ends once the animal reaches the bottom of the pole. Adapted from BioRender and not drawn to scale

The pole test was used to assess motor dysfunction in mice. Each mouse was placed facing upwards to the top of a 55 cm-long, 1 cm-diameter pole (Fig. 3). Parameters such as the latency to turn downwards (time the mouse takes to make a 180° turn), the time to descend, and the total time spent on the pole were measured manually. The test was carried out over two consecutive days: three training trials on the first day and three final trials on the second day. The final trials were recorded with a front-view camera for subsequent manual analysis, and mouse performances were then averaged.

### Immunoassays

#### Tissue Processing

Animals were deeply anesthetised and perfused transcardially with saline and then 4% paraformaldehyde (PFA) in 0.1 M phosphate-buffered saline (PBS). Brains were post-fixed in PFA at 4 °C for 48 h and stored in a cryoprotectant solution. They were then coronally sectioned at 40 μm using a freezing microtome and kept at − 20 °C until use.

#### Immunofluorescence

Immunofluorescence assays were performed to study the overexpression of α-syn, its aggregation through phosphorylation (p-syn), the diffusion capacity in the brain of the wild-type and phosphorylated forms of α-syn, and p-syn’s co-expression with dopaminergic neurons (stained with tyrosine hydroxylase, TH). This technique was also used to analyse the astroglial (glial fibrillary acidic protein, GFAP) and microglial (ionised calcium binding adaptor molecule type 1, IBA-1) activation in the dopaminergic nigrostriatal pathway. α-Syn immunoreactivity was assessed using a human-specific α-syn antibody to selectively detect AAV-mediated human A53T α-syn expression, thereby avoiding confounding detection of endogenous murine α-syn.

The specificity of the primary antibodies used has been verified in various articles (Delic et al. 2018; Jakes et al. 1999) and on vendor websites.

Free-floating sections were washed 3 times (5 min each) in 0.1 M PBS. To block non-specific binding sites, sections were then incubated for 2 h at room temperature (RT) with agitation in a blocking solution consisting of 5% normal goat serum (NGS) (Sigma-Aldrich, ref. S26-LITER) and 0.5% Triton X-100 in 0.1 M PBS. Sections were then incubated overnight at 4 °C with the corresponding primary antibodies (mouse anti-α-syn, 1:10000, Abcam, ref. AB27766; rabbit anti-p-syn, 1:1000, Abcam, ref. AB51253; mouse anti-TH, 1:1000, Merck Millipore, ref. MAB5280; rabbit anti-IBA-1, 1:1000, Fujifilm Wako, ref. 019-19741; rabbit anti-GFAP, 1:500, Dako, ref. Z0334) in 0.1 M PBS containing 5% NGS and 0.5% Triton X-100. The next day, after 3 5-minute washes in 0.1 M PBS, sections were incubated for 2 h with the corresponding fluorescent secondary antibodies (goat anti-mouse Alexa Fluor 594, 1:400, Invitrogen, ref. A11005; goat anti-rabbit Alexa Fluor 594, 1:400, Invitrogen, ref. A11012; goat anti-rabbit Alexa Fluor 488, 1:400, Invitrogen, ref. A11008; goat anti-mouse Alexa Fluor 488, 1:400, Invitrogen, ref. A11001) in a 0.1 M PBS solution containing 1% NGS and 0.5% Triton X-100. Subsequently, the last 3 5-minute washes were performed in 0.1 M PBS. Finally, the sections were mounted on Superfrost slides (EprediaTM, 12302108) and coverslipped with a mounting medium (Vectashield).

#### Immunohistochemistry

This technique was applied to evaluate α-syn and p-syn overexpression and dopaminergic neuron integrity via TH staining. α-Syn immunostaining was performed using a mouse monoclonal antibody (Abcam AB27766, clone LB509) that selectively recognises human α-syn and does not detect endogenous murine α-syn, allowing specific assessment of AAV-mediated transgene expression. The pSer129 α-syn antibody (Abcam AB51253; 1:1000) has been widely validated for detecting α-syn-associated pathology in multiple AAV-α-syn rodent models (Ip et al. 2017; Koprich et al. 2010; Van der Perren et al. 2015) and was confirmed in our study by its specific labelling of discrete neuronal inclusions in AAV-A53T α-syn-injected animals but not in empty vector controls.

The specificity of the primary antibodies used has been verified in various articles (Delic et al. 2018; Jakes et al. 1999) and on vendor websites.

These immunostains were performed on free-floating sections that were initially washed 3 times (5 min each) in 0.1 M PBS. Endogenous peroxidases were quenched with 3% (α-syn and p-syn) or 30% (TH) H_2_O_2_ and 10% methanol in 0.1 M PBS for 30 min at RT. After three washes with 0.1 M PBS to block non-specific binding sites, sections were incubated for 1 h at RT in a blocking solution containing 5% normal horse serum (NHS) and 0.5% Triton X-100 in 0.1 M PBS. Sections were then incubated overnight at 4 °C in the same blocking solution together with the corresponding primary antibody (mouse anti-α-syn, 1:10000, Abcam, ref. AB27766; rabbit anti-p-syn, 1:1000, Abcam, ref. AB51253; rabbit anti-TH, 1:1000, Merck Millipore, ref. AB152). The next day, after 3 5-minute washes in 0.1 M PBS, sections were incubated for 2 h at RT with the corresponding biotinylated secondary antibody (horse anti-mouse IgG, 1:200, Vector Laboratories, ref. BP-2000-50; horse anti-rabbit IgG, 1:200, Vector Laboratories, ref. BA1100-1.5) using 2.5% NHS and 0.5% Triton X-100 in 0.1 M PBS. After three washes in 0.1 M PBS were done, and sections were incubated with an avidin-biotin-peroxidase complex (ABC kit, 1:200, Vector Laboratories, ref. PK-6100) and 0.5% Triton X-100 in 0.1 M PBS for 1 h at RT. Peroxidase activity was visualised using the chromogen 3,3′-diaminobenzidine (DAB, Sigma-Aldrich, ref. D5637) and H_2_O_2_. Finally, sections were mounted, air dried for 48 h, cleared in xylene for at least 2 h and coverslipped with DPX synthetic resin mounting medium (Sigma-Aldrich, ref. 06522).

### Quantification Procedures

#### Optical Densitometry Analysis

Striatal, *nucleus accumbens* (NAcc) and nigral sections of TH; and striatal and nigral α-syn stainings were optically digitised at a resolution of 6400 ppp using an EPSON Perfection V700 scanner and EPSON Scan software (version 3.9.2.2). α-Syn^+^ and TH^+^ fibre densitometry analyses were performed using the ImageJ software (version 2.9.0, NIH). At the 60 days time point, 9–15 striatal, 4–6 NAcc and 4–7 nigral sections of TH per animal were used, and 6–7 striatal, 3–4 nigral sections of α-syn stainings were used. At the 120 days time point, 4–6 striatal, 2–4 NAcc and 5–7 nigral sections of TH per animal were used, and 4–6 striatal, 1–3 nigral sections of α-syn stainings were used. The measured values were normalised for non-specific background staining by subtracting the values obtained from the *corpus callosum* or the basal part of the cerebral peduncle.

### Stereological Quantification of Dopaminergic Neurons in the Substantia Nigra Pars Compacta

Quantification was performed with an Olympus BX51 microscope and the optical fractionator method via Mercator software (Explora-Nova, La Rochelle, France). This method uses systematic, random sampling of 3D sections to estimate cell numbers. SNc cell counting was done between Bregma − 2.70 mm and − 3.88 mm, referencing the Paxinos and Franklin atlas (Paxinos and Franklin 2004).

Cells were counted at 40x magnification within a 4x delineated area, following stereological rules: neurons inside the inclusion lines or touching them were counted, while those on exclusion lines were not. The total number of cells was estimated using the formula: N = ΣQ × (1/ssf) × (1/asf) × (1/hsf), where Q is the number of counted cells. The section sampling factor (ssf) was 1/5, the counting frame size of 50 × 50 μm with a separation between dissectors of 60 μm, and the height sampling fraction (hsf) of 95%. Approximately 5–8 sections per animal were analysed. The volume was estimated using the Cavalieri method to calculate neuron density (cells/mm³) for each group.

#### Macro-based Quantification of Axonal Swellings

Images were captured using an Olympus BX61 microscope connected to an Olympus U-CMAD3 digital camera. A 60x oil objective was used, with a z-stack interval of 1 μm covering the full section thickness. One field of view in the striatum was imaged per hemisphere per coronal Sect.  10 AAV-α-syn animals (5 males, 5 females) were analysed at each time point, with 6 sections imaged per animal, totalling 12 image stacks per animal. No images were taken in empty vector animals as they do not present axonal swellings.

Axonal swellings in the striatum were quantified using ImageJ (version 1.8.0), following an automated workflow adapted from Quintino et al. (2022). Briefly, a *Batch* of images was generated to undergo the macro-based processing to quantify axonal swellings. The process included converting z-stacks into 8-bit grayscale images, inverting background contrast, and applying several image enhancement steps: rolling ball background subtraction, pixel subtraction, median smoothing, and contrast enhancement. Thresholding was then applied to identify structures.

Only particles larger than 0.53 μm² and with a minimum voxel number of 5 were classified as axonal swellings. For each animal, the total number and median volume of axonal swellings were calculated.

#### Axonal Degeneration Index Calculation

To quantify the extent and temporal progression of striatal axonal damage, we developed an Axonal Degeneration Index (ADI) that combines two complementary measures: striatal TH optical density, reflecting fibre integrity, and the number of α-syn-positive axonal swellings, indicative of ongoing axonal degeneration. Because axonal swellings follow a non-linear, inverted U-shaped trajectory—starting low in early stages, peaking as axons become damaged, and declining as fibres fragment—using either measure alone can misrepresent the degeneration stage. To integrate these signals, TH levels and swelling counts were z-score normalised per time point, and animals were classified into early, intermediate, or advanced degeneration stages based on thresholds derived from the data distribution and the expected dynamics of TH loss and swelling formation. Early-stage axons (score 1) were defined by high TH (z > 0.3) and low swelling (z < − 0.5), intermediate-stage axons (score 2) by moderate TH (z between − 0.3 and 0.3) and variable swelling, and advanced-stage axons (score 3) by low TH (z < − 0.3) with either high (z > 0.5) or low (z < − 0.5) swelling depending on active degeneration or completed fibre loss, respectively. This approach provides a sensitive, data-driven measure of axonal pathology progression. The distributions of TH optical density and axonal swelling count data for all animals, together with their relationship to ADI scores are shown in Supplementary Fig. S1.

#### Glial Activation

To assess glial activation, GFAP^+^ and IBA-1^+^ cells were analysed in the striatum and SN. Images were acquired with a Zeiss Axioskop fluorescence microscope (40× objective) connected to a Nikon DS-Qi1 mono camera. In each hemisphere, 3 images were taken in the striatum (dorsal, medial, ventral) and 3 in the *substantia nigra* (SN) (medial, central, lateral).

Astrocyte and microglia activation was semi-quantified using ImageJ (version 1.8.0) by calculating the GFAP^+^ and IBA-1^+^ areas relative to the total area (mm²), respectively. To ensure consistent analysis, all images were batch processed using the same settings: background removal with the “Subtract Background” tool (rolling ball radius: 20 pixels), Gaussian Blur (sigma: 2), and automatic brightness/contrast adjustment. The “Moments” thresholding method was applied to select GFAP^+^ and IBA-1^+^ areas. Processed images were saved, and results were exported to a spreadsheet for further analysis.

### Statistical Analysis

Statistical analyses were conducted in R (version 4.4.1). Data are reported as mean ± SD. Behavioural and morphological data were analysed using two-way ANOVA. The main factors were α-syn group and sex, and an interaction term (α-syn group × sex) was included in the model. Each time point was analysed independently. Model assumptions of normality and homoscedasticity were assessed for all variables; behavioural variables were log-transformed when necessary, and all variables ultimately satisfied these assumptions prior to analysis. For the wire hang test, analyses were additionally performed using ANCOVA with body weight included as a covariate to control for its potential influence on performance. Comparisons of ADI between A53T α-syn males and females were performed using the Mann–Whitney U test, due to the presence of only two groups and a non-normal distribution of the variable as indicated by the Shapiro-Wilk test. The results were presented graphically using GraphPad Prism (version 8.0.1; GraphPad Software Inc., USA).

## Results

### Alpha-Synuclein Diffusion and Overexpression. Co-localisation with Dopaminergic Neurons

At 60 days post-surgery, mice injected with AAV-A53T α-syn showed higher α-syn levels than controls in both the striatum (F_(1,12)_ = 31.1, *p* < 0.001) and SN (F_(1,12)_ = 7.8, *p* = 0.016), while this increase appeared more pronounced in males than in females, the difference did not reach statistical significance (Fig. S2). This indicates that α-syn overexpression was successfully induced in both sexes at the early time point. At 120 days post-surgery, α-syn levels remained elevated in AAV-A53T mice compared with controls in both regions (striatum: F_(1,15)_ = 47.0, *p* < 0.001; SN: F_(1,15)_ = 13.3, *p* = 0.002; Fig. S2). At this later time point, however, females displayed a clearer increase in α-syn levels than males (striatum: F_(1,15)_ = 7.1, *p* = 0.018; SN: F_(1,15)_ = 5.5, *p* = 0.033), revealing a more pronounced progression of α-syn accumulation in females over time (Fig. S2).

To determine whether SNc dopaminergic neurons accumulated phosphorylated α-syn (pSer129; p-syn), double immunofluorescence for TH and p-syn was performed. Co-localisation of p-syn within TH-positive neurons confirmed the presence of α-syn-associated pathology in nigral dopaminergic cells (Fig. S3). P-syn immunoreactivity was restricted to the injection site in the SN and was consistently observed in both sexes at all examined time points (Figs. S4, S5). It is important to note that, in the context of AAV-mediated α-syn overexpression models, the presence of α-syn immunoreactivity—particularly phosphorylated α-syn at Ser129—should be interpreted as an indicator of α-syn-associated pathology rather than definitive evidence of self-propagating misfolded species.

Given the anatomical proximity of the ventral tegmental area (VTA) to the SNc and its dopaminergic nature, this region was analysed to assess potential spread of the viral vector and the specificity of the model. α-Syn expression was detected in the VTA; however, no p-syn staining was observed (Fig. S6a), and there was no loss of TH-positive fibres in the nucleus accumbens (NAcc; Fig. S6b). These findings indicate that, despite some α-syn expression in neighbouring dopaminergic nuclei, α-syn-associated pathology and dopaminergic fibre loss remain largely confined to the targeted nigrostriatal pathway.

### Motor Impairment in Mutant A53T Alpha-Synuclein Mice

Body weight was monitored from the day of surgery up to 120 days post-surgery and increased steadily in all experimental groups, consistent with normal growth and with no differences between treatments or sexes (Fig. S7).

**Fig. 4:**
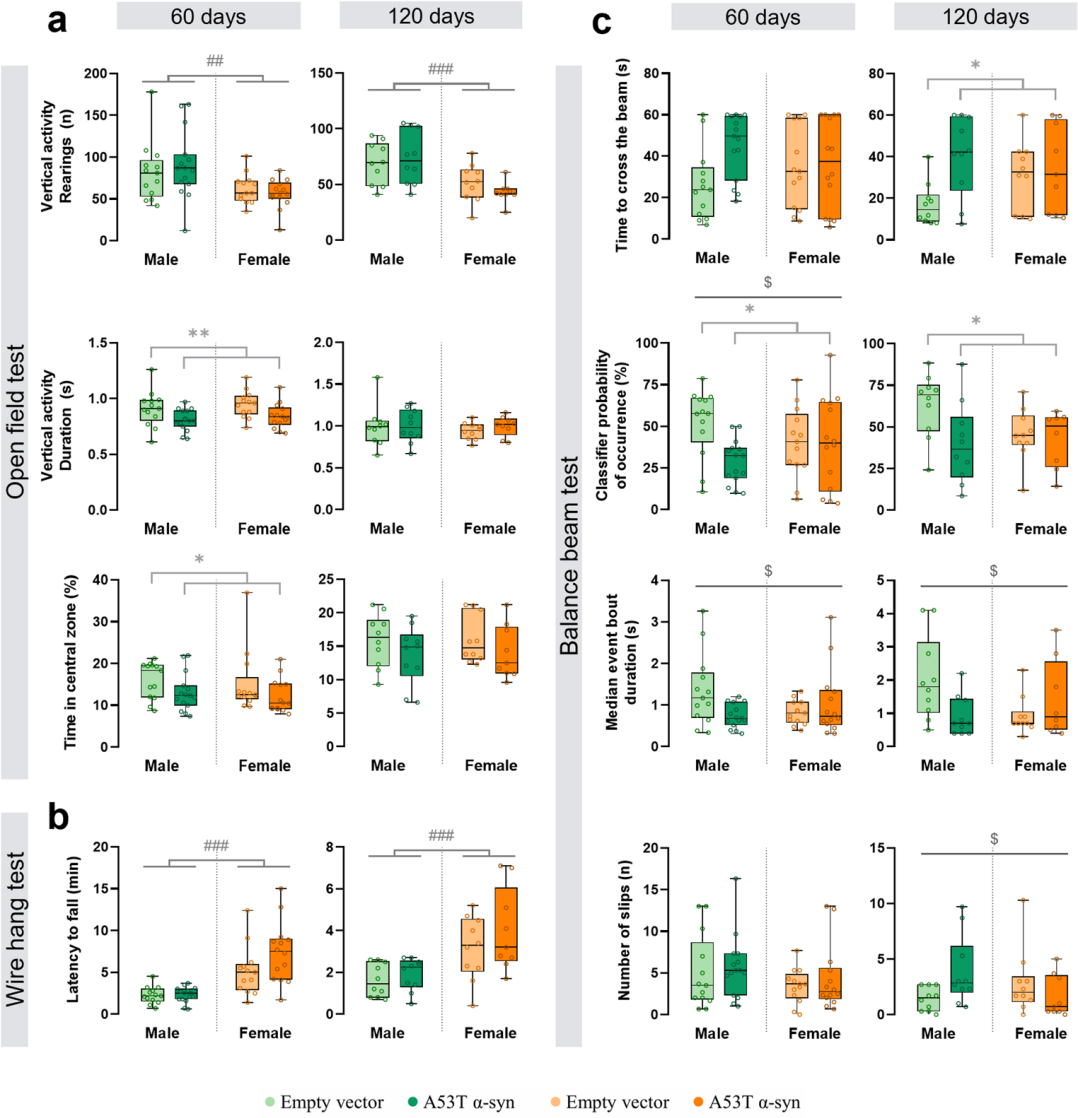
Motor impairment in mutant A53T alpha-synuclein (α-syn) mice. Mice performed the open field (**a**), wire hang (**b**) and balance beam tests (**c**) at 60 and 120 days post-surgery. Statistical significance was determined using two-way ANOVA, with significance levels represented by: * for the α-syn group effect *p < 0.05; **p < 0.01; ^#^ the sex effect ^##^*p* < 0.01; ^###^*p* < 0.001; and ^$^ denoting the interaction effect (α-syn group × sex) with ^$^*p* < 0.05. *60 days*: male empty vector, *n* = 13; male α-syn, *n* = 15; female empty vector, *n* = 13; female α-syn, *n* = 14; *120 days*: male empty vector, *n* = 10; male α-syn, *n* = 10; female empty vector, *n* = 10; female α-syn, *n* = 9

In the open field test, total locomotor activity was not altered by A53T α-syn overexpression (Table S1). However, at 60 days post-injection, α-syn mice reduced the rearing time (vertical activity duration, F_(1,51)_ = 10.1, *p* = 0.002) and spent less time in the central zone of the arena (F_(1,51)_ = 5.2, *p* = 0.027), indicating early changes in exploratory behaviour and anxiety-like responses (Fig. 4a). At both 60 and 120 days, male mice exhibited a greater number of rearings compared to female mice (F_(1,51)_ = 11.3, *p* = 0.001, and F_(1,35)_ = 17.0, *p* < 0.001, respectively; Fig. 4a). At 60 days, A53T α-syn modified maximum speed in a sex-dependent manner: speed tended to decrease in males and increase in females, suggesting opposite effects on locomotor dynamics in each sex but without reaching significance (Table S1).

In the wire hang test, females exhibited an increased latency to fall from the grid at both 60 and 120 days post-surgery (F_(1,51)_ = 48.5, *p* < 0.001, and F_(1,35)_ = 17.7, *p* < 0.001, respectively; Fig. 4b), meaning that female mice showed longer hanging times than males, even after adjusting for body weight.

Automated analysis of the balance beam test revealed subtle but consistent impairments in motor function and balance in A53T α-syn mice at both time points. Across 60 and 120 days, α-syn mice showed reduced walking along the beam compared with empty vector mice, reflected by a lower probability of being classified as “walking” (F_(1,51)_ = 4.6, *p* = 0.036, and F_(1,34)_ = 4.4, *p* = 0.044, respectively; Fig. 4c). Additionally, at 120 days, α-syn mice tended to fail the task more often (Table S1), receiving the default maximum crossing time (60s) without having reached the finish line. They also took longer to cross the beam, reaching significance at 120 days only (F_(1,35)_ = 6.2, *p* = 0.018; Fig. 4c). These motor impairments tended to be more marked in males, indicating that the slowing of beam traversal and decrease in walking were predominantly a male-biased effect. Similarly, α-syn males exhibited a more fragmented walking pattern along the beam, reflected by the increase in the number of bouts, nearly reaching significance at 120 days (Table S1).

A more detailed analysis of sex differences showed that A53T α-syn overexpression affected several parameters predominantly in males. At 60 days, α-syn markedly reduced the percentage of time spent walking on the beam in males but not in females (classifier probability of occurrence, F_(1,51)_ = 4.2, *p* = 0.046; Fig. 4c). Similarly, α-syn reduced the duration of uninterrupted walking episodes in males while tending to increase it in females, at both 60 days (median event bout duration: F_(1,51)_ = 6.3, *p* = 0.015; Fig. 4c) and 120 days post-surgery (median event bout duration: F_(1,34)_ = 6.9, *p* = 0.013; Fig. 4c; and mean event bout duration: F_(1,34)_ = 5.2, *p* = 0.029; Table S1). At 60 days, A53T α-syn overexpression tended to increase the time spent immobile/frozen in males while reducing it in females (mean event bout interval duration, Table S1). At 120 days, α-syn mice had a tendency to spend more time immobile on the beam, independent of sex (mean event bout interval duration, Table S1).

Manual analysis of the balance beam test revealed that A53T α-syn overexpression increased the number of hind paw slips in males while reducing it in females, reaching significance at 120 days only (F_(1,35)_ = 5.8, *p* = 0.021; Fig. 4c).

Performance in the pole test was not affected by A53T α-syn overexpression at any time point (Table S1).

Overall, these findings indicate that A53T α-syn overexpression does not affect total activity or gross motor performance. Nonetheless, subtle and task-specific motor alterations emerge, particularly in balance and fine locomotor control, and these changes develop differently in males and females, with a greater functional impact in males over time.

### Dopaminergic Degeneration in the Nigrostriatal Pathway

To assess whether A53T α-syn overexpression induces dopaminergic degeneration in the nigrostriatal pathway and whether this varies by sex, cell density of TH^+^ soma in the SNc and optical density of TH^+^ fibres in the striatum and *substantia nigra pars reticulata* (SNr) were quantified in male and female mice.

**Fig. 5:**
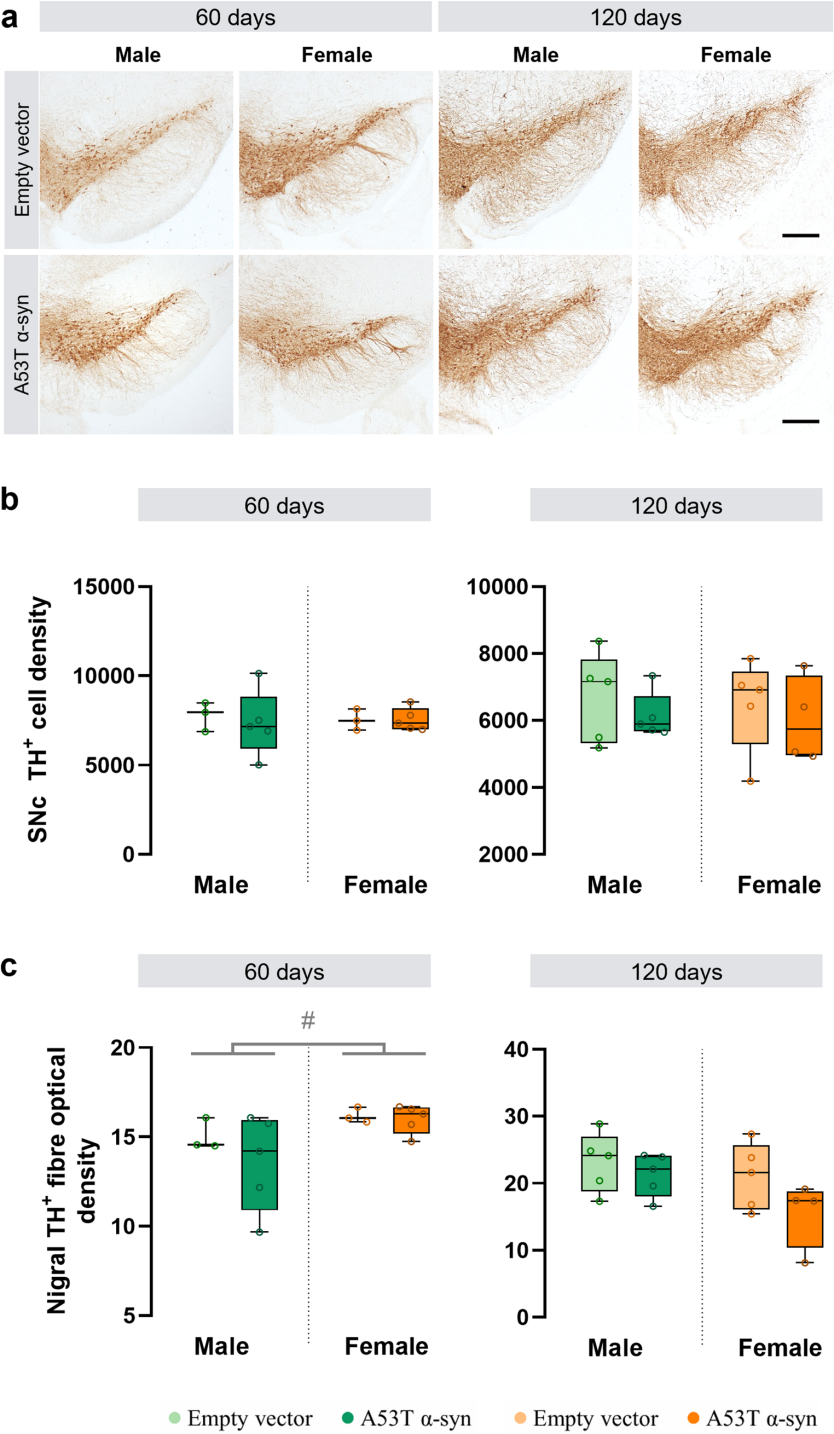
Dopaminergic cell density analysis in the *substantia nigra pars compacta* (SNc) and tyrosine hydroxylase-positive (TH^+^) dendritic density analysis in the *substantia nigra pars reticulata* (SNr) in male and female A53T alpha-synuclein (α-syn) and empty vector animals. **a** Immunohistochemistry for TH in the *substantia nigra* of representative samples from empty vector and α-syn groups, 60 and 120 days after virus administration. Scale bar: 300 μm. **b** Stereological quantification of TH^+^ cell density in the SNc at 60 and 120 days post-surgery. **c** Quantification of the optical density of dopaminergic fibres in the SNr at 60 and 120 days post-surgery. Statistical significance was determined using two-way ANOVA, with significance levels represented by: # for the sex effect #*p* < 0.05. *60 days*: male empty vector, *n* = 3; male α-syn, *n* = 5; female empty vector, *n* = 3; female α-syn, *n* = 5; *120 days*: male empty vector, *n* = 5; male α-syn, *n* = 5; female empty vector, *n* = 5; female α-syn, *n* = 4

**Fig. 6:**
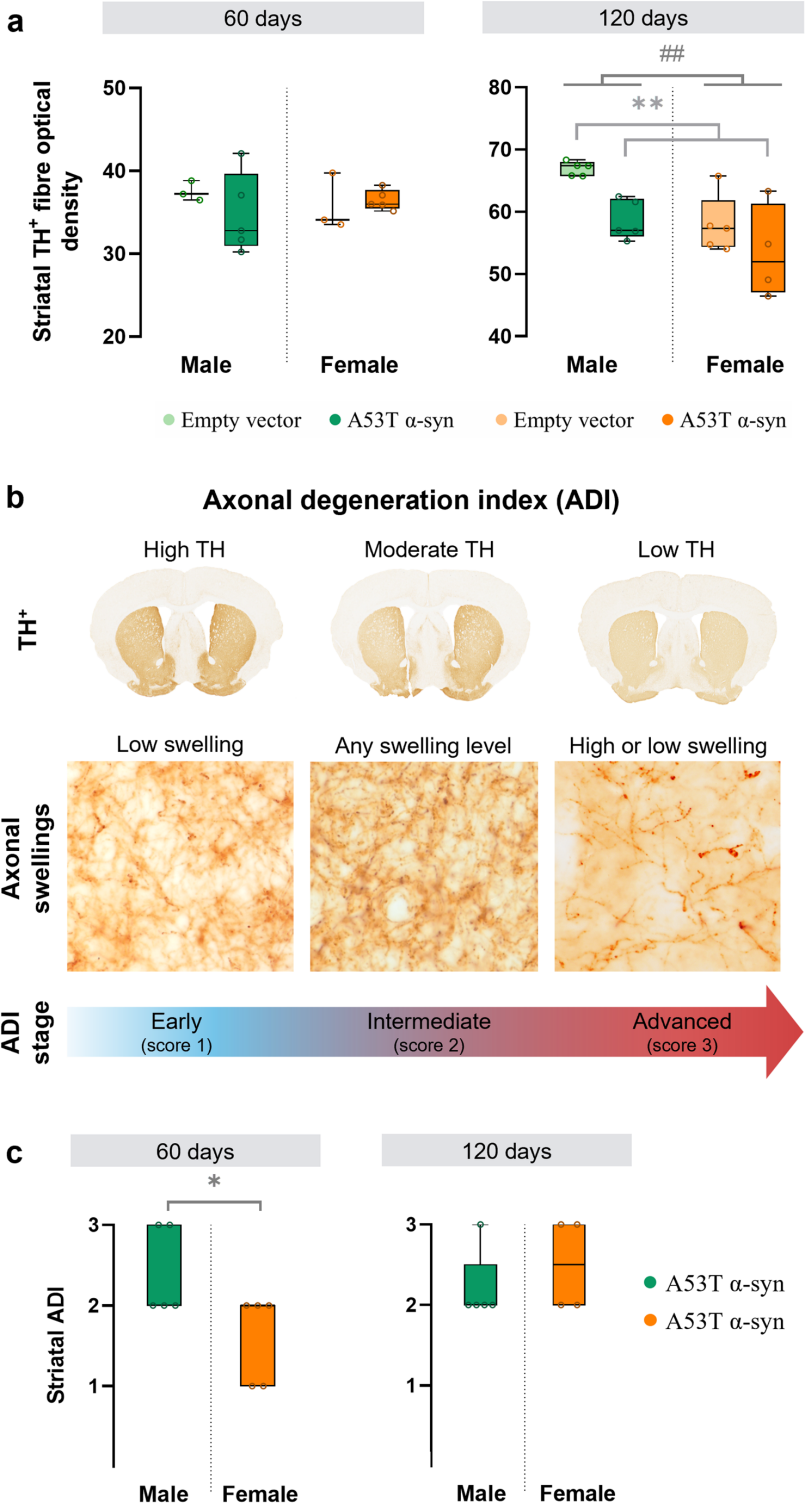
Sex-dependent pattern of striatal degeneration in A53T alpha-synuclein (α-syn) mice. **a** Quantification of the optical density of dopaminergic fibres in the striatum in male and female A53T α-syn and empty vector animals. Dopaminergic neurons were positive for tyrosine hydroxylase (TH^+^). Statistical significance was determined using two-way ANOVA, with significance levels represented by: * for the α-syn group effect **p < 0.01; and ^#^ the sex effect ^##^*p* < 0.01. **b** The Axonal Degeneration Index (ADI) was calculated to quantify the extent and progression of axonal damage in the striatum of α-syn mice. This index was derived by combining two complementary measures: striatal TH optical density and the number of striatal axonal swellings detected via immunohistochemistry against α-syn. **c** Striatal ADI in male and female A53T α-syn mice. Statistical significance was determined using Mann-Whitney U test, with *p < 0.05. *60 days*: male empty vector, *n* = 3; male α-syn, *n* = 5; female empty vector, *n* = 3; female α-syn, *n* = 5; *120 days*: male empty vector, *n* = 5; male α-syn, *n* = 5; female empty vector, *n* = 5; female α-syn, *n* = 4

AAV-A53T α-syn injection did not reduce TH^+^ soma density in the SNc (Fig. 5a, b) or TH^+^ dendritic density in the SNr (Fig. 5c) at either time point. However, at 60 days post-surgery, the density of nigral TH^+^ dendrites was higher in females than in males (F_(1,12)_ = 5.5, *p* = 0.037; Fig. 5c). Striatal TH optical density decreased after α-syn overexpression, reaching significance only at 120 days post-surgery (F_(1,15)_ = 8.9, *p* = 0.009; Fig. 6a), with similar reductions in males and females, driven by α-syn overexpression (Fig. 6a). At 120 days, female mice were also found to have lower striatal TH^+^ dendrites compared to males (F_(1,15)_ = 12.4, *p* = 0.003; Fig. 6a).

TH optical density alone may underestimate early axonopathy, as TH^+^ fibres often exhibit swelling and fragmentation before complete loss of TH expression. These changes can peak prior to fibre loss, masking degeneration when swellings are prominent. To address this, A53T α-syn mice were classified into three stages of degeneration based on combined striatal TH optical density and axonal swelling counts (Fig. 6b). Using this integrated approach, males showed more advanced axonal degeneration (2.6 ± 0.6) than females (1.6 ± 0.6) at 60 days post-surgery (Shapiro Wilk test, W = 0.833, *p* = 0.039; Mann-Whitney U test, W = 22, *p* = 0.041; Fig. 6c). By 120 days, degeneration levels converged between sexes (males 2.2 ± 0.8; females 2.5 ± 0.6; Mann–Whitney U test, W = 8, *p* = 0.687), indicating a faster initial progression of axonopathy in males.

### Neuroinflammation in the Nigrostriatal Pathway

To determine whether A53T α-syn overexpression induces glial activation, astrocytes (GFAP-positive) and microglia (IBA-1-positive) were quantified in the striatum and SN of male and female mice.

**Fig. 7:**
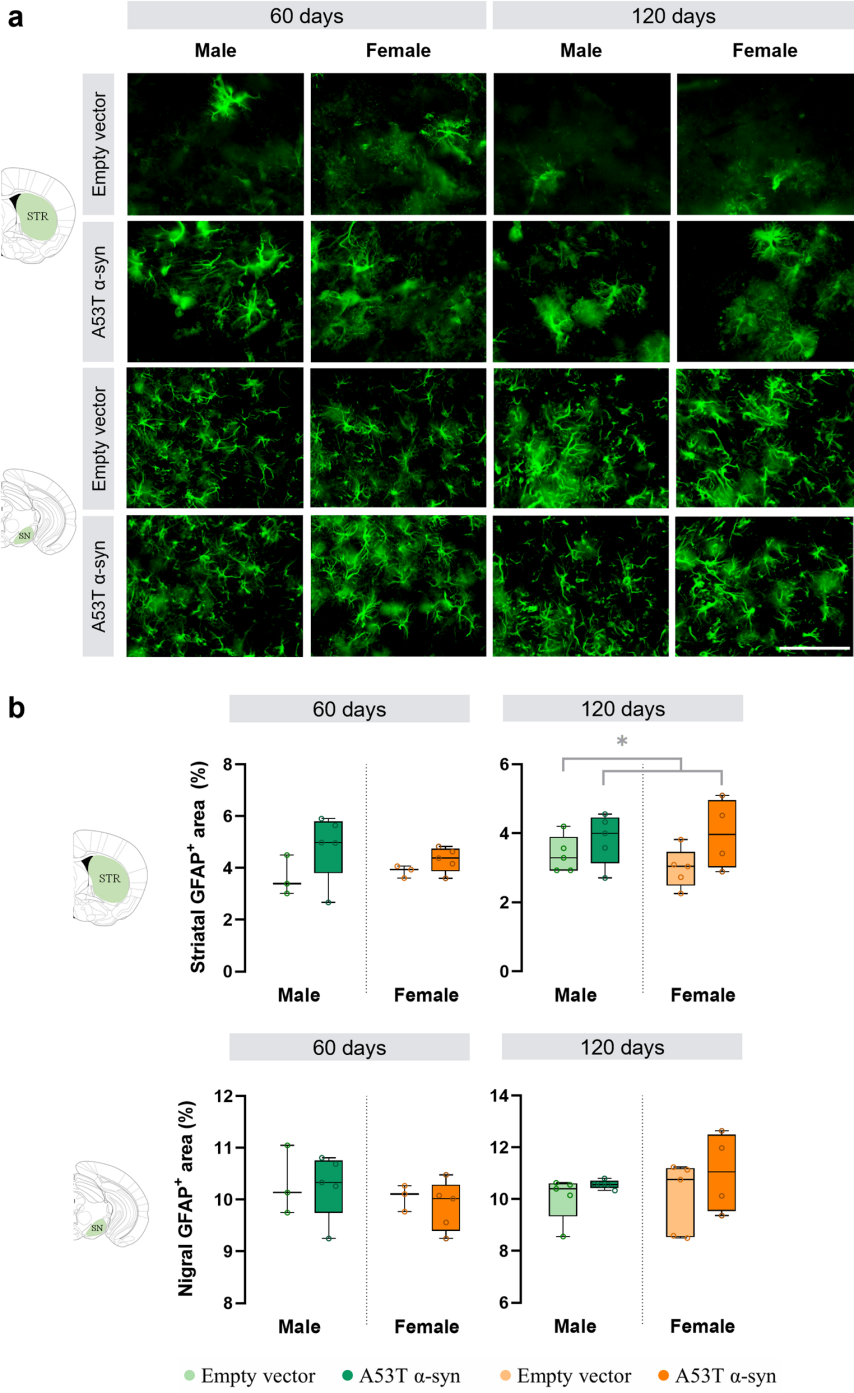
Astrocytic activation in the nigrostriatal pathway. **a** Representative images showing glial fibrillary acidic protein (GFAP) immunofluorescence in the striatum and substantia nigra of empty vector and α-synuclein (α-syn) animals at 60 and 120 days post-surgery. Scale bar: 80 μm. **b** Quantification of GFAP expression in both regions at each time point. Statistical significance was determined using two-way ANOVA, with significance levels represented by: * for the α-syn group effect *p < 0.05. *60 days*: male empty vector, *n* = 3; male α-syn, *n* = 5; female empty vector, *n* = 3; female α-syn, *n* = 5; *120 days*: male empty vector, *n* = 5; male α-syn, *n* = 5; female empty vector, *n* = 5; female α-syn, *n* = 4

α-Syn mice showed markedly increased astrocytic activation in the striatum compared with empty vector controls, reaching significance at 120 days post-surgery (F_(1,15)_ = 4.8, *p* = 0.045; Fig. 7). This elevated astroglial response confirms neuroinflammation in the striatal compartment. Representative images illustrate this astrocytic activation in the striatum and SN (Fig. 7). In contrast, AAV-A53T α-syn injection did not induce significant astrocytic activation in the SN at either time point (Fig. 7). Microglial activation was also unaffected in both the striatum and SN across all groups and time points (Fig. S8).

No sex differences were detected in astrocytic or microglial activation, indicating that α-syn-driven neuroinflammation occurs independently of sex in this model.

## Discussion

In this study, we examined the consequences of mutant A53T α-syn overexpression in a mouse model, focusing on the progression of pathological changes and their sex-specific manifestations. The key pathological domains assessed included motor function, dopaminergic fibre integrity, axonal pathology, and astrocytic activation. Our findings demonstrate that while both sexes eventually reach comparable levels of neurodegeneration, the timing and trajectory of these processes differ significantly between males and females, particularly at early stages. The observed subtle sex-specific effects should be considered preliminary and warrant confirmation in larger cohorts.

### Behavioural Alterations and Phenotypic Relevance

Behaviourally, A53T α-syn overexpression induced early motor and affective alterations detectable at 60 days post-injection. Anxiety-like behaviour in the open field test was measured by central avoidance (Bunnell et al. 2025; Deng et al. 2021; Nuber et al. 2013). Mice showed increased anxiety-like behaviour consistent with clinical observations in PD, where anxiety frequently co-occurs with motor symptoms and may precede overt motor manifestations (Coakeley et al. 2014). Despite preserved gross motor function in the pole test, subtle impairments in motor coordination were detected in the balance beam test, supporting the notion that balance and gait disturbances can emerge before more global motor deficits (Costa et al. 2020; Sirabella et al. 2018). These findings suggest a selective early deficit in fine motor coordination and balance rather than generalised bradykinesia, reflecting early-stage or prodromal PD phenotypes. Sex-specific differences were evident across several behavioural domains. In the wire hang test, females exhibited significantly longer latencies to fall than males at both 60 and 120 days. Because females weighed less than males, and body mass is known to influence hanging performance, we explicitly controlled for body weight using ANCOVA. Although body weight significantly contributed to performance at both time points, the sex effect remained highly significant after adjustment, indicating that differences in body mass alone do not fully account for the observed phenotype. These findings suggest that additional factors, potentially including sex-dependent motivational, affective, or anxiety-related components, may also influence wire hang performance, and warrant cautious interpretation of this measure as a purely physiological readout. Importantly, our automated analysis of balance beam walking behaviour using DeepLabCut and SimBA (Bidgood et al. 2024), revealed subtle but consistent motor deficits in males, including reduced active walking time compared with females. This supports a distinct temporal progression of behavioural alterations between sexes that might be overlooked by traditional manual scoring.

From a phenotypic standpoint, these behavioural differences likely reflect sex-specific functional thresholds for the emergence of motor symptoms. Males appear to express functional impairment earlier, whereas females may remain behaviourally compensated for longer, despite ongoing α-syn–induced pathology. This dissociation between early pathological burden and delayed behavioural expression is consistent with the concept of compensatory reserve within the nigrostriatal system, whereby early neurodegenerative changes do not immediately translate into overt motor deficits.

Existing literature on sex differences in early motor phenotypes in PD remains inconclusive, with some clinical studies suggesting milder motor symptoms in men (Solla et al. 2012), whereas others report less severe impairment in women (Kang et al. 2022; Kim et al. 2018). Reduced vulnerability in females has been attributed to higher physiological dopamine levels in the nigrostriatal pathway (Lee et al. 2015) and the neuroprotective actions of oestrogens via anti-inflammatory, antioxidant, anti-apoptotic, and anti-aggregation mechanisms (Rajsombath et al. 2019; Rugbjerg et al. 2013; Thadathil et al. 2021). However, sex-specific data in early-stage PD are limited, highlighting the novelty of these findings.

### Axonal Pathology as an Early Substrate of Sex-Dependent Functional Changes

Morphologically, A53T α-syn overexpression induced early axonal alterations in α-syn mice. Striatal TH optical density showed only a modest decline at 60 days, with a significant reduction emerging at 120 days. Axonal alterations have been previously reported in experimental PD models based on AAV-mediated overexpression of α-syn. Van der Perren et al. (2015) described the presence of swollen neurites in the striatum of rats following an AAV-A53T α-syn injection into the SN. Likewise, Phan et al. (2017) reported axonal swellings in female rats injected into the SNc with wild-type α-syn.

However, early axonal swelling and fragmentation can confound isolated TH optical density measurements by preserving TH immunoreactivity despite structural disruption. By combining TH optical density and axonal swelling counts into a composite axonal degeneration index (using z-scored variables and stratification into early, intermediate, and advanced stages), the data indicate a fluctuating temporal pattern, with male A53T α-syn mice exhibiting earlier neurodegenerative changes as early as 60 days post-injection. These findings support a distinct progression of neurodegeneration between sexes. Notably, both males and females displayed TH levels in the SNr and dopaminergic cell densities in SNc comparable to empty vector mice at these time points, suggesting that axonal pathology precedes measurable loss of dopaminergic terminals or cell bodies.

Our findings highlight sex-specific differences in the early pattern of striatal neurodegeneration that are not detectable by TH optical density alone. Consistent with these observations, previous studies in A53T α-syn mice reported SNc dopaminergic neuron loss only at later stages (around 12 months), with males showing earlier gross motor impairment (Costa et al. 2020; Sirabella et al. 2018). *Postmortem* analyses in PD patients similarly indicate that, at motor symptoms onset, loss of dopaminergic terminals in the striatum or putamen exceeds neuronal loss in the SNc (Bernheimer et al. 1973; Kish et al. 1988; Scherman et al. 1989). Although 60–80% of SNc dopamine neurons are lost by late disease stages (Fearnley and Lees 1991; Pakkenberg et al. 1991), depletion of dopaminergic markers in the striatum is even more pronounced, supporting the concept that axonal degeneration precedes somatic neuronal loss (Burke and O’Malley 2013). Human neuroimaging studies further show that dopaminergic neurodegeneration initially manifests more prominently in axonal projections (Caminiti et al. 2017), in line with the present results.

Overall, our findings provide preclinical support for an axonopathy-first model, while adding novel evidence of sex-dependent variation in this retrograde degenerative process. It is important to note that AAV-mediated α-syn overexpression represents a targeted approach primarily affecting the nigrostriatal system, whereas transgenic models typically overexpress α-syn throughout the brain and may not preferentially target dopaminergic neurons. A study by Games et al. (2013) investigated axonopathy in transgenic mice overexpressing human wild-type α-syn, and found abundant clusters of dystrophic neurites in the striatum, midbrain among other brain areas, similar to neuritic dystrophy observed in PD patients. In addition, in a transgenic mouse model overexpressing α-syn, dopaminergic axonal pathology in the striatum was detected in the absence of overt dopaminergic neuronal loss in the SNc (Tofaris et al. 2006). Other studies using transgenic A53T or A30P mice have reported sex-specific effects, including earlier motor deficits and more pronounced axonal pathology in males (Costa et al. 2020; Sirabella et al. 2018). Our findings are largely consistent with these observations, indicating that male mice display a faster progression of axonal degeneration and subtle motor impairments, while females exhibit delayed onset. This highlights that sex differences are robust across multiple experimental approaches to α-syn overexpression, and underscores the importance of considering sex as a biological variable when interpreting neuropathological and behavioural outcomes in both AAV and transgenic models.

Importantly, the earlier emergence of axonal pathology in males temporally parallels the earlier detection of subtle motor coordination deficits, supporting a close structure–function relationship in which accelerated axonopathy likely contributes to earlier phenotypic expression of motor impairment. A study involving male rats that overexpressed the A53T α-syn revealed a significant correlation between striatal dopaminergic loss and motor impairment (Musacchio et al. 2022). In contrast, females display a relative dissociation between early axonal pathology and functional outcome, remaining behaviourally compensated despite ongoing structural degeneration.

Interestingly, females showed higher α-syn levels at 120 days despite later onset of axonal degeneration and behavioural deficits, suggesting greater resilience in females. Such sex-dependent compensation has been proposed in both clinical and experimental PD and may be supported by higher physiological dopamine levels in the nigrostriatal pathway (Lee et al. 2015), as well as the neuroprotective actions of oestrogens via anti-inflammatory, antioxidant, anti-apoptotic, and anti-aggregation mechanisms (Rajsombath et al. 2019; Rugbjerg et al. 2013; Thadathil et al. 2021). Consistent with this interpretation, Lamontagne-Proulx et al. (2023) demonstrated that the cessation of oestrous cycling is associated with the loss of female-specific neuroprotection in α-syn–related pathology, supporting the notion that ovarian hormones actively modulate disease vulnerability rather than merely delaying onset.

Although direct gonadectomy studies in AAV-mediated α-syn overexpression models remain limited, converging experimental evidence supports a causal role for oestrogens. In a recent study using the MPTP model, which induces α-syn accumulation in addition to dopaminergic toxicity, oophorectomy abolished female-specific resistance to striatal dopamine depletion, motor deficits, and α-syn pathology. Pharmacological activation of the G-protein–coupled oestrogen receptor reversed these effects (Liang et al. 2025). Together, these findings suggest that ovarian hormones modulate both the structural (axonopathy) and functional (motor coordination) progression of early α-syn toxicity, allowing females to remain behaviourally compensated for longer despite ongoing pathology. Future studies incorporating oestrous cycle monitoring or ovariectomy paradigms will be essential to directly test this hypothesis in AAV-A53T α-syn models.

### Glial Responses and Inflammatory Considerations

Astrocytic activation was evident in the striatum at 120 days, temporally aligning with striatal dopaminergic degeneration. No significant astrocytic changes were detected in the SN, which may reflect a later onset of glial responses in regions relatively spared from early dopaminergic loss. In parallel, the similar astrocytic activation observed in the SNc between AAV-A53T α-syn and empty vector groups argues against a major contribution of differential viral load to the observed neuropathology. Previous reports of SN astrogliosis in 12-month-old A53T mice (Costa et al. 2020; Sirabella et al. 2018) support this interpretation. Although no sex differences in astrocytic activation were detected at the examined time points, it remains possible that earlier assessments might reveal divergent glial responses between males and females. Although IBA1-positive microglia was present at 60 and 120 days post-surgery, the decrease observed at 120 days suggests that microglial reactivity in the SN is transient and likely reflects a general post-surgical response rather than a specific response to human α-syn expression. Future studies assessing microglial morphology (ramified *versus* amoeboid) could provide a more sensitive measure of the state and involvement of microglia in α-syn clearance and neuroinflammation, even in the absence of overall changes in IBA-1 area. In any case, the potential for temporally and sexually divergent glial responses warrants further investigation.

### Strengths and Limitations

This study has several strengths. First, integrating TH optical density with axonal swelling counts into a composite degeneration index enabled sensitive detection of distinct striatal axonal degeneration profiles between sexes that might be missed by traditional single-parameter approaches. Second, the use of machine learning-based automated behavioural analysis (DeepLabCut and SimBA) allowed high-resolution detection of subtle motor impairments, reducing observer bias and improving reproducibility. Together, these methodological advances provide a more refined characterisation of early disease stages, which is critical for understanding sex-specific trajectories in PD progression.

Several limitations should also be acknowledged. First, experimenters were not blinded to all the experimental procedures, such as to manual behavioural scoring (wire hang and pole tests) and tissue processing. The experimenters were aware of the experimental groups at the time of data acquisition due to logistical and practical constraints. Nevertheless, all procedures were conducted in accordance with strict, predefined protocols, and the outcome measures were based on objective, quantitative criteria. Importantly, several analyses were conducted using an automated approach to minimise subjectivity (open field and balance beam tests, and axonal swelling counts). In addition, the stereological approach used to quantify the number of neurons in the SNc is an unbiased method. Therefore, while the lack of blinding should be acknowledged, we believe that it did not materially affect the validity or interpretation of the results. Second, the small and unbalanced sample size in control subgroups (*n* = 3 per sex) reduces statistical power and limits the interpretability of interaction effects. Sex differences should therefore be interpreted cautiously. Third, because of technical constraints, only two time points (60 and 120 days post-injection) were analysed, limiting the temporal resolution of early dynamics. Including earlier time points would help clarify the onset of glial responses and better define sex-specific differences in their evolution, whereas later assessments could reveal whether SNc neuronal loss diverges further between sexes. Fourth, AAV-mediated models inherently show variability in viral transduction efficiency; although animals with insufficient viral spread were excluded, this remains a general limitation. Furthermore, α-syn immunostaining was performed using a human-specific antibody (Abcam AB27766, clone LB509), which allowed selective detection of AAV-mediated transgene expression but did not permit assessment of endogenous murine α-syn or conformationally misfolded species. The use of well-characterised, species- and conformation-specific antibodies (e.g., D37A6, MJFR1, MJFR14) represents an important direction for future studies to more precisely dissect α-syn aggregation dynamics and propagation mechanisms. Finally, AAV-mediated models inherently show variability in viral transduction efficiency; although animals with insufficient viral spread were excluded, this remains a general limitation. Nonetheless, this model is well suited to studying early axonopathy, which is highly relevant translationally, as axonal degeneration often precedes neuronal death and may represent a therapeutic window during which disease progression could be delayed. Future work should also address the potential neuroprotective role of oestrogen, which may underlie sex differences in vulnerability. Moreover, quantifying striatal dopamine levels would provide a more complete picture of functional impairment and its relationship to structural changes. Future experiments would benefit from a statistical design explicitly modelling incorporating time, sex, and treatment factors simultaneously.

## Conclusion

In summary, these findings show that A53T α-syn overexpression induces progressive dopaminergic axonopathy accompanied by subtle but robust sex-specific differences in the temporal profiles of motor alterations and neuroinflammation. Males exhibit a faster progression of axonal pathology and earlier, though subtle, motor changes compared with females. These results underscore the importance of considering sex as a biological variable in PD models and support the development of sex-specific therapeutic strategies targeting early disease stages.

## Supplementary Information

Below is the link to the electronic supplementary material.

Supplementary Material 1

## Data Availability

Data supporting this study are available on the OSF Repository at: 10.17605/OSF.IO/SZYEF). For further inquiries, please contact the corresponding author. Preprint available in BioRxiV (10.1101/2025.07.30.667616).
