# Supplementary material for: Sex-Dependent Dynamics of Behavioural and Neuropathological Changes in an A53T Alpha-Synuclein Mouse Model of Parkinson’s Disease
Source: Cell Mol Neurobiol. 2026 Mar 4;46:70. doi: 10.1007/s10571-026-01707-9 (PMC13003041; doi:10.1007/s10571-026-01707-9)
Supplement: Supplementary file 1 — Supplementary Material 1 [file 10571_2026_1707_MOESM1_ESM.pdf]

SUPPLEMENTARY INFORMATION

Sex-dependent dynamics of behavioural and neuropathological changes in an A53T alpha-synuclein mouse model of Parkinson's disease

Maider Zubelzu[1,2], Raphaelle Bidgood[1*], Ane Murueta-Goyena[2,3], Jose Angel Ruiz-Ortega[1,2], Jose Vicente Lafuente[2,3] and Teresa Morera-Herreras[1,2]

[1]LanCE-Neuropharm Research Group, Department of Pharmacology, Faculty of Medicine and Nursery, University of the Basque Country (EHU), Leioa, Spain.

[2]Neurodegenerative Diseases Group, Biobizkaia Health Research Institute, Barakaldo, Bizkaia, Spain.

[3]LanCE-Neuropharm Research Group, Department of Neurosciences, Faculty of Medicine and Nursery, University of the Basque Country (EHU), Leioa, Spain.

CONTENTS:

Supplementary Tables (1)

Supplementary Figures (8)

* Corresponding author:

Raphaelle Bidgood
LanCE-Neuropharm Research Group, Department of Pharmacology, Faculty of Medicine and Nursing, University of the Basque Country (UPV/EHU)
E-mail address: rbidgood001@ikasle.ehu.eus
Postal address: Department of Pharmacology, Faculty of Medicine and Nursing, University of the Basque Country (EHU), Barrio Sarriena s/n, 48940-Leioa, Spain
Telephone number: +34 94 601 3316
ORCID number: 0009-0005-8190-1461

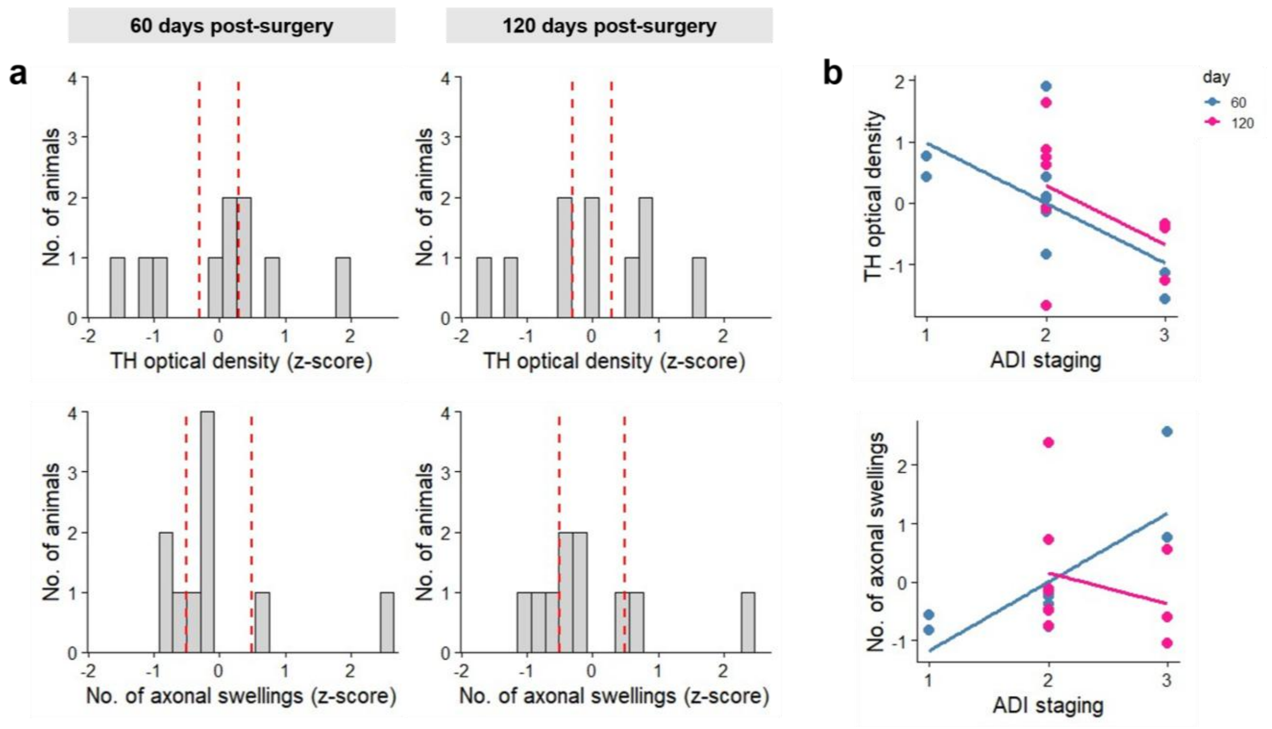

Fig. S1 Distribution of standardised pathology measures and their relationship to axonal degeneration index (ADI) staging. (a) Histograms showing the empirical distributions of z-scored striatal tyrosine hydroxylase (TH) optical density and z-scored axonal swelling counts across animals, with dashed lines indicating the thresholds used for ADI staging (TH: −0.3 and 0.3; axonal swelling: −0.5 and 0.5). Thresholds were selected based on the observed data distributions to define low, intermediate, and high pathology ranges while maintaining an approximately balanced representation of animals across stages and preserving biologically interpretable transitions. **(b)** Scatterplots depicting the relationship between ADI stage and each underlying measure (TH optical density and axonal swelling burden). Each point represents an individual animal, with time points color-coded. 60 days: male α-syn, n = 5; female α-syn, n = 5; 120 days: male α-syn, n = 5; female α-syn, n = 4

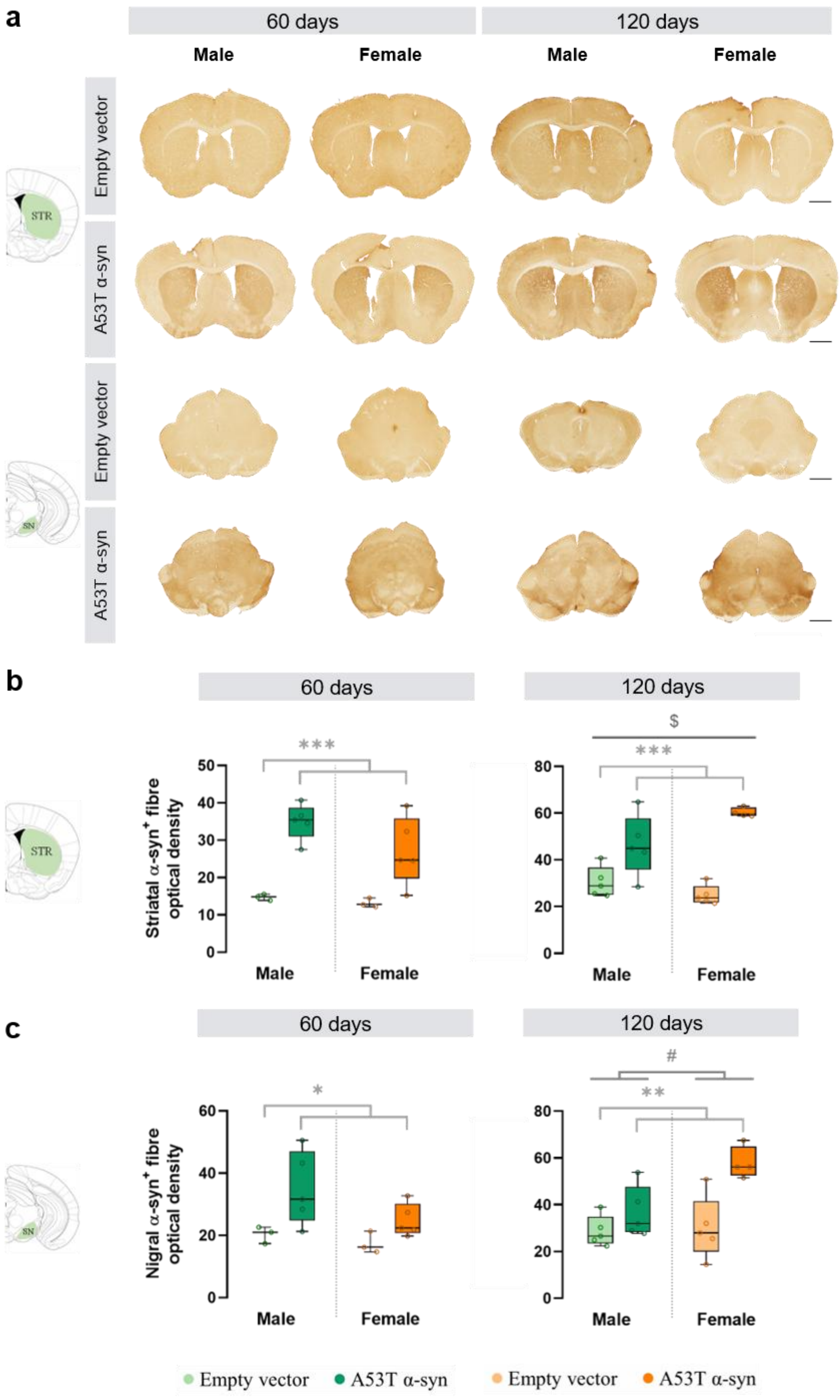

a
60 days
120 days
Male
Female
Male
Female
Empty vector
A53T α-syn
Empty vector
A53T α-syn
STR
SN
b
60 days
120 days
Striatal α-syn+ fibre optical density
Male
Female
$
***
c
Nigral α-syn+ fibre optical density
#
**
*
Empty vector
A53T α-syn
Empty vector
A53T α-syn

Fig. S2 Alpha-synuclein (α-syn) overexpression in male and female A53T α-syn and empty vector animals. (a) Representative images of striatal and nigral α-syn overexpression in empty vector and α-syn animals at 60 and 120 days after surgery. Scale bar: 1000 µm. Quantification of the optical density of α-syn^{+} fibres in the striatum **(b)** and *substantia nigra* (SN) **(c)** in male and female mice at both experimental time points: 60 and 120 days post-injection. Statistical significance was determined using two-way ANOVA, with significance levels represented by: * for the α-syn group effect *$p<0.05$; **$p<0.01$; ***$p<0.001$; # the sex effect #$p<0.05$; and $ denoting the interaction effect (α-syn group × sex) with \$$p<0.05$. 60 days: male empty vector, n = 3; male α-syn, n = 5; female empty vector, n = 3; female α-syn, n = 5; 120 days: male empty vector, n = 5; male α-syn, n = 5; female empty vector, n = 5; female α-syn, n = 4

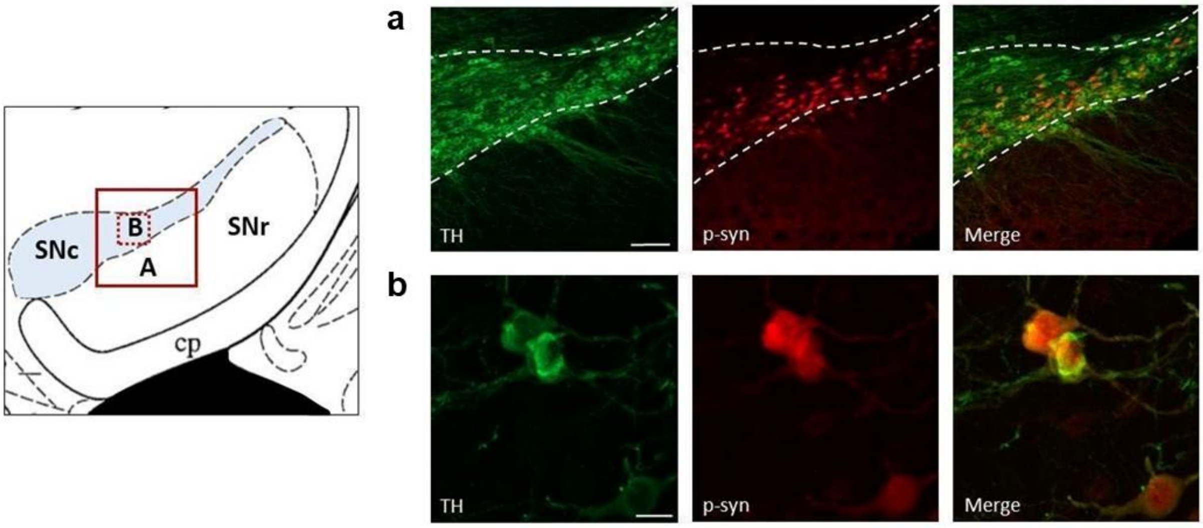

Fig. S3 Representative images of the *substantia nigra pars compacta* (SNc) showing the co-expression of phosphorylated alpha-synuclein (p-syn) and tyrosine hydroxylase (TH). Positive labelling is shown for TH (in green), p-syn (in red) and their co-expression (in yellow) in dopaminergic neurons of the SNc. **(a)** Scale bar: 100 µm (objective 10x). **(b)** Scale bar: 15 µm (objective 63x)

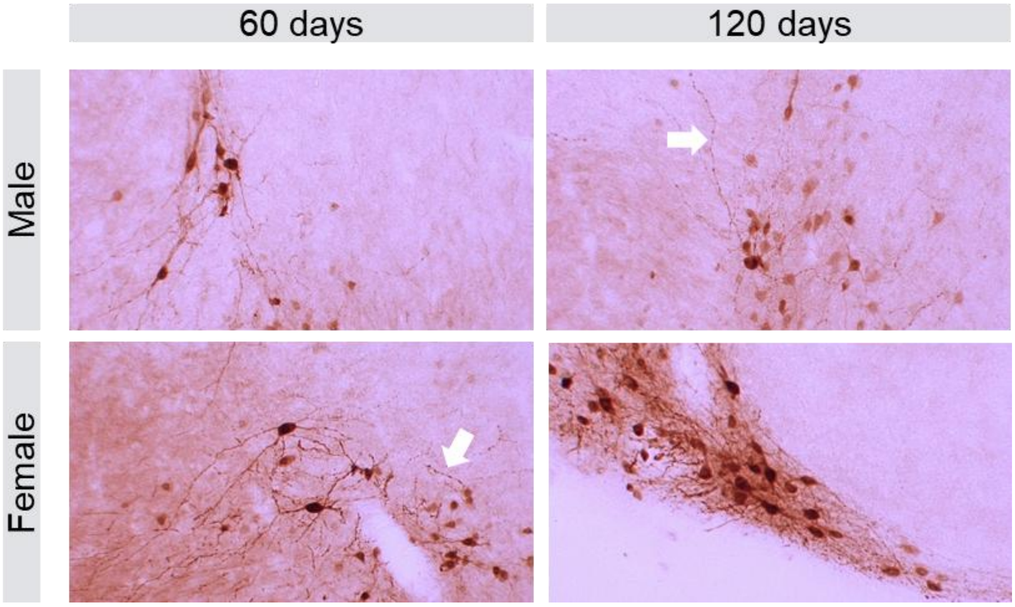

Fig. S4 Expression of phosphorylated alpha-synuclein (p-syn) in the *substantia nigra* of male (top) and female (bottom) mice at 60 and 120 days after surgery. White arrows indicate the axonal swellings observed in these p-syn-expressing neurons

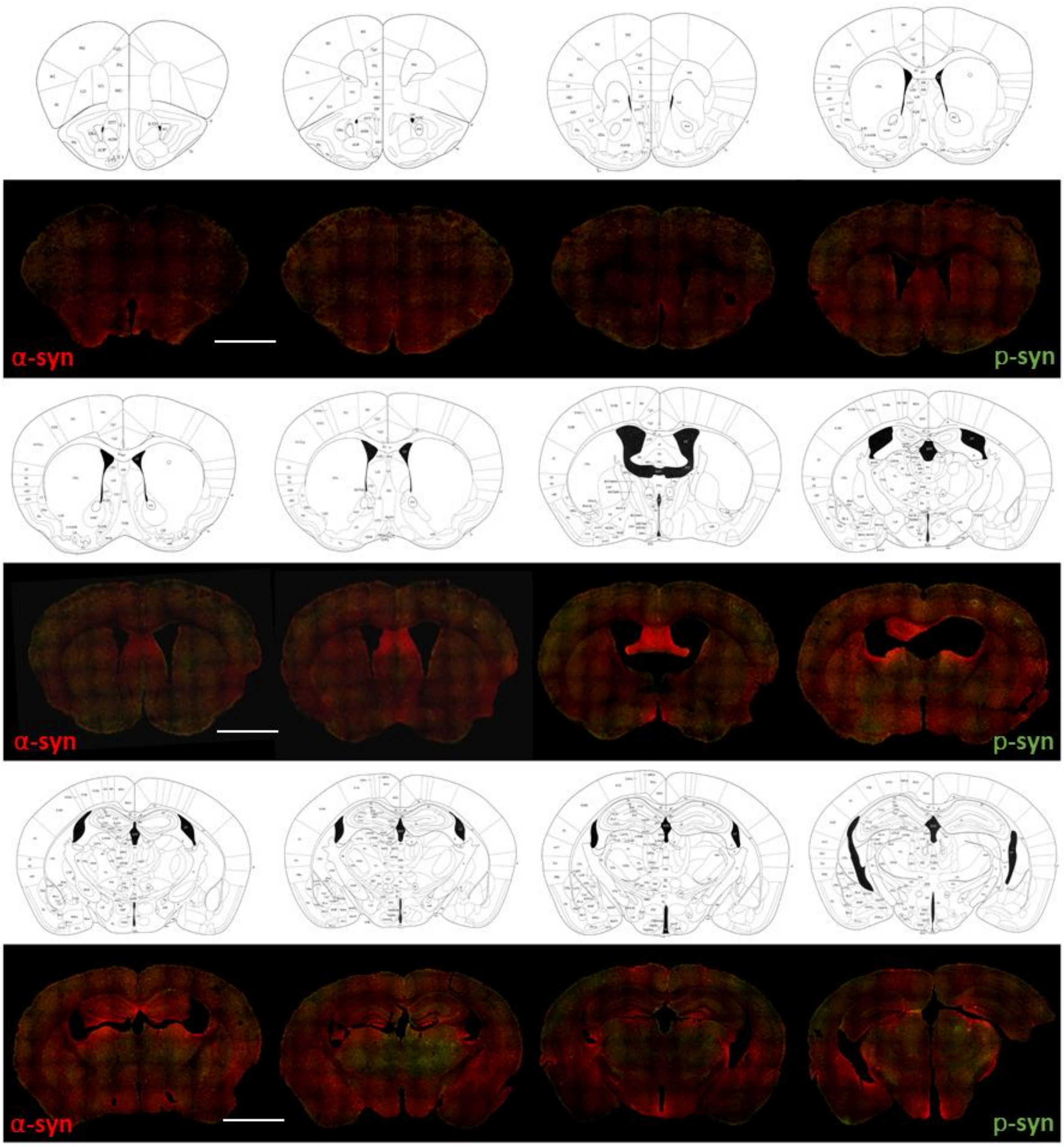
α-syn
p-syn
α-syn
p-syn
α-syn
p-syn

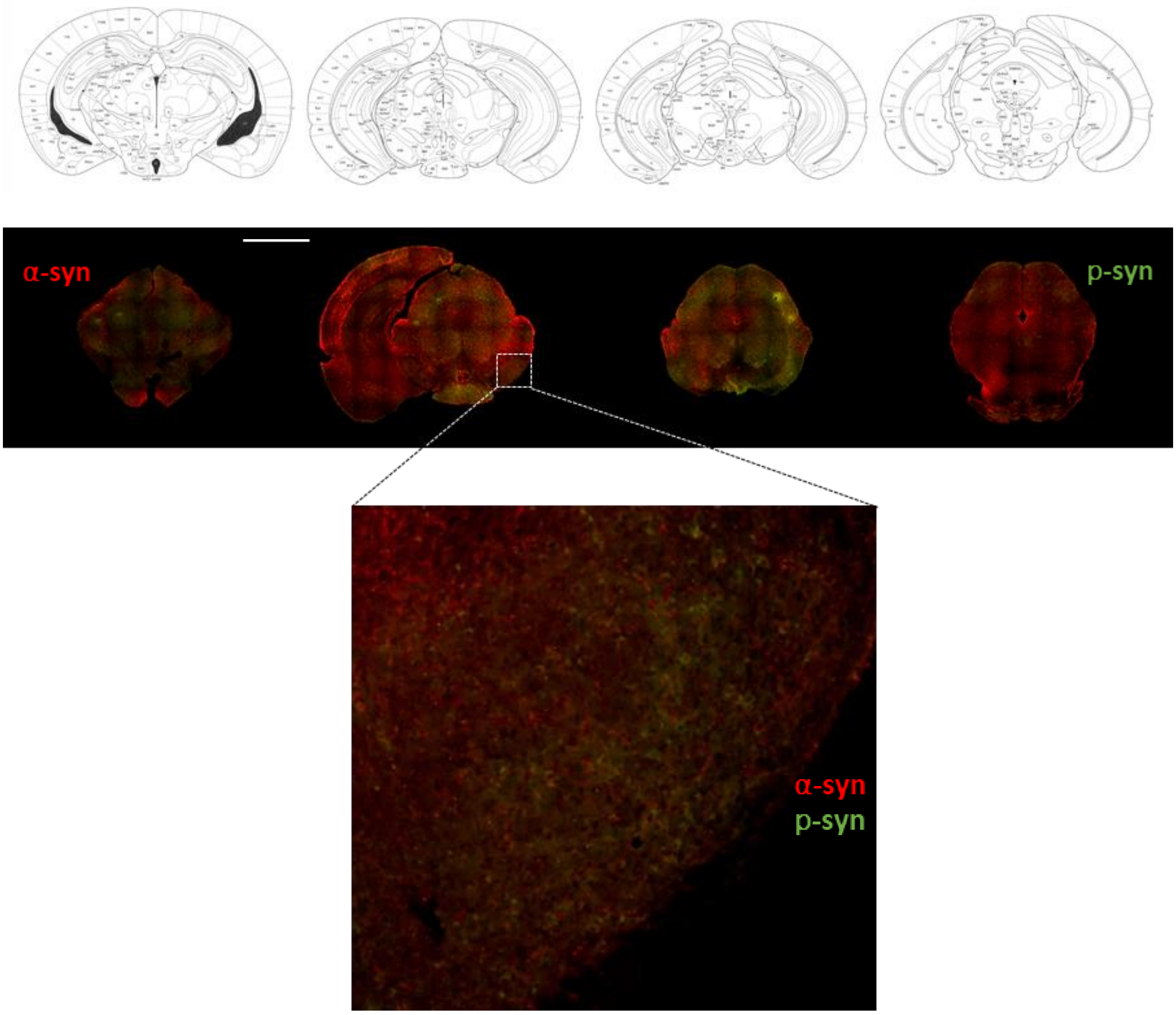

Fig. S5 Serial immunofluorescence for alpha-synuclein (α-syn, in red) and phosphorylated alpha-synuclein (p-syn, in green). Serial brain sections from a male animal 120 days after surgery. Scale bar: 2000 μm

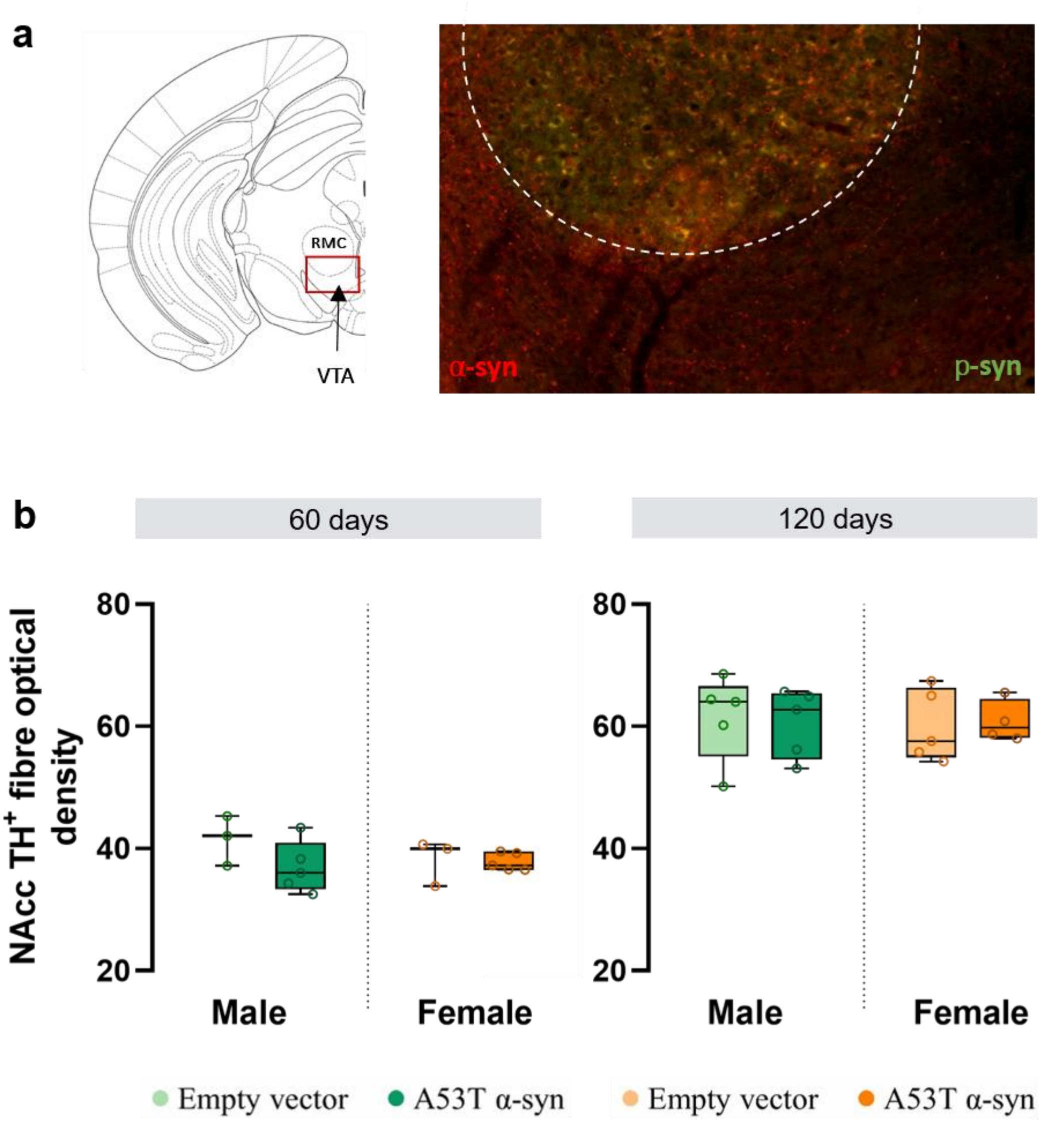

Fig. S6 Preserved dopaminergic projections from the ventral tegmental area (VTA) to the *nucleus accumbens* (NAcc). (a) Double immunofluorescence for alpha-synuclein (α-syn, in red) and phosphorylated alpha-synuclein (p-syn, in green) of a male animal 120 days after surgery. **(b)** Quantification of the optical density of dopaminergic fibres in the NAcc in male and female A53T α-syn and empty vector animals. Dopaminergic neurons were positive for tyrosine hydroxylase (TH^+). No differences in dopaminergic degeneration were observed in the NAcc at either 60 days or 120 days post-surgery. 60 days: male empty vector, n = 3; male α-syn, n = 5; female empty vector, n = 3; female α-syn, n = 5; 120 days: male empty vector, n = 5; male α-syn, n = 5; female empty vector, n = 5; female α-syn, n = 4

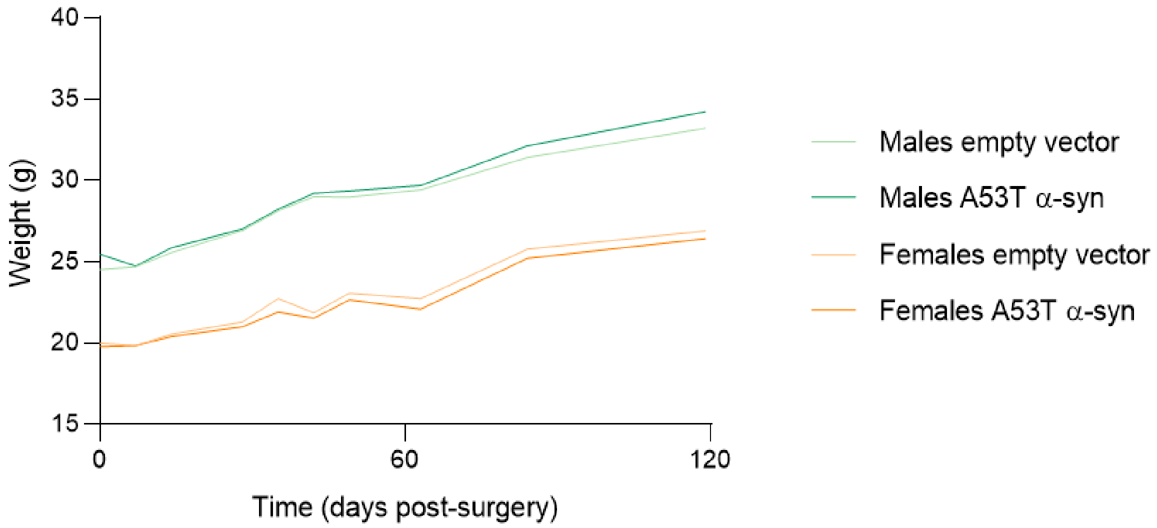

Fig. S7 Body weight of animals monitored from the day of surgery until 120 days post-surgery. The body weight of mice increased steadily over this period in all experimental groups (empty vector and A53T alpha-synuclein (α-syn) mice), consistent with normal growth

Table S1 Results summary of other parameters analysed in the behavioural tests performed in male and female A53T alpha-synuclein (α-syn) and empty vector animals.

Mice performed the tests at 60 and 120 days post-surgery. For each behavioural test parameter, the number of mice (n), mean ± standard deviation (SD) and statistical results are reported. A two-way ANOVA was carried out to analyse possible group (G), sex (S) and/or α-syn group × sex interaction (G×S) effects. Statistical significance was determined using two-way ANOVA, with significance levels represented by: $ denoting the interaction effect (G×S) with $p<0.05 and close to significance values highlighted in bold

				60 days post-injection			120 days post-injection		
Behavioural tests		Experimental group		n	Mean ± SD	Statistics	n	Mean ± SD	Statistics
Open field test	Total activity (cm)	Male	Empty vector	13	2616 ± 415.9	G: $F_{(1,51)}$=0.213; p=0.646<br>S: $F_{(1,51)}$=0.156; p=0.695<br>G×S: $F_{(1,51)}$=0.288; p=0.594	10	2559 ± 336.6	G: $F_{(1,35)}$=0.767; p=0.387<br>S: $F_{(1,35)}$=0.158; p=0.694<br>G×S: $F_{(1,35)}$=0.292; p=0.593
			A53T α-syn	15	2512 ± 380.7		10	2516 ± 500.3	
		Female	Empty vector	13	2598 ± 449.1		10	2674 ± 233.4	
			A53T α-syn	14	2607 ± 325.5		9	2496 ± 445.4	
	Stereotyped movements (n)	Male	Empty vector	13	210.2 ± 33.18	G: $F_{(1,51)}$=0.094; p=0.760<br>S: $F_{(1,51)}$=0.240; p=0.627<br>G×S: $F_{(1,51)}$=0.556; p=0.459	10	230.3 ± 27.54	G: $F_{(1,35)}$=1.495; p=0.230<br>S: $F_{(1,35)}$=0.815; p=0.373<br>G×S: $F_{(1,35)}$=0.108; p=0.745
			A53T α-syn	15	200.3 ± 34.08		10	214.7 ± 42.14	
		Female	Empty vector	13	207.4 ± 39.62		10	236.3 ± 26.00	
			A53T α-syn	14	211.8 ± 35.04		9	227.4 ± 29.82	
	Locomotion (n)	Male	Empty vector	13	2406 ± 394.0	G: $F_{(1,51)}$=0.211; p=0.648<br>S: $F_{(1,51)}$=0.137; p=0.712	10	2328 ± 316.3	G: $F_{(1,35)}$=0.672; p=0.418<br>S: $F_{(1,35)}$=0.117; p=0.735
			A53T α-syn	15	2311 ± 361.0		10	2301 ± 465.7	

		Female	Empty vector	13	2390 ± 417.9	<u>G×S:</u> $F_{(1,51)}$=0.247; p=0.622	10	2438 ± 236.1	<u>G×S:</u> $F_{(1,35)}$=0.359; p=0.553
			A53T α-syn	14	2396 ± 312.2		9	2269 ± 422.9	
	**Maximum speed (cm/s)**	Male	Empty vector	13	32.45 ± 11.64	<u>G:</u> $F_{(1,51)}$=0.855; p=0.360<br><u>S:</u> $F_{(1,51)}$=0.101; p=0.752<br><u>G×S:</u> $F_{(1,51)}$=3.213; **p=0.079**	10	27.92 ± 2.953	<u>G:</u> $F_{(1,35)}$=0.036; p=0.851<br><u>S:</u> $F_{(1,35)}$=1.378; p=0.248<br><u>G×S:</u> $F_{(1,35)}$=0.064; p=0.802
			A53T α-syn	15	27.27 ± 4.131		10	28.26 ± 2.633	
		Female	Empty vector	13	29.42 ± 5.131		10	27.14 ± 1.698	
			A53T α-syn	14	31.16 ± 5.678		9	27.06 ± 3.039	
	**Mean speed (cm/s)**	Male	Empty vector	13	7.492 ± 1.362	<u>G:</u> $F_{(1,51)}$=0.113; p=0.738<br><u>S:</u> $F_{(1,51)}$=0.583; p=0.449<br><u>G×S:</u> $F_{(1,51)}$=0.082; p=0.776	10	7.440 ± 1.150	<u>G:</u> $F_{(1,35)}$=0.595; p=0.446<br><u>S:</u> $F_{(1,35)}$=0.006; p=0.940<br><u>G×S:</u> $F_{(1,35)}$=0.228; p=0.636
			A53T α-syn	15	7.260 ± 1.438		10	7.310 ± 1.563	
		Female	Empty vector	13	7.669 ± 1.387		10	7.670 ± 0.8097	
			A53T α-syn	14	7.657 ± 1.491		9	7.133 ± 1.661	
	**Distance travelled (cm)**	Male	Empty vector	13	4498 ± 827.1	<u>G:</u> $F_{(1,51)}$=0.115; p=0.736<br><u>S:</u> $F_{(1,51)}$=0.553; p=0.460<br><u>G×S:</u> $F_{(1,51)}$=0.079; p=0.780	10	4457 ± 679.9	<u>G:</u> $F_{(1,35)}$=0.561; p=0.459<br><u>S:</u> $F_{(1,35)}$=0.018; p=0.894<br><u>G×S:</u> $F_{(1,35)}$=0.211; p=0.649
			A53T α-syn	15	4358 ± 866.8		10	4381 ± 944.9	
		Female	Empty vector	13	4601 ± 831.1		10	4606 ± 485.8	
			A53T α-syn	14	4592 ± 893.2		9	4295 ± 1000	
	**Resting time (s)**	Male	Empty vector	13	76.03 ± 43.75	<u>G:</u> $F_{(1,51)}$=0.174; p=0.679<br><u>S:</u> $F_{(1,51)}$=0.003; p=0.953<br><u>G×S:</u> $F_{(1,51)}$=0.048; p=0.827	10	75.90 ± 32.82	<u>G:</u> $F_{(1,35)}$=1.981; p=0.168<br><u>S:</u> $F_{(1,35)}$=0.182; p=0.672<br><u>G×S:</u> $F_{(1,35)}$=0.309; p=0.582
			A53T α-syn	15	82.63 ± 34.31		10	86.90 ± 48.69	
		Female	Empty vector	13	77.83 ± 45.42		10	63.44 ± 25.88	
			A53T α-syn	14	79.84 ± 30.50		9	88.76 ± 49.23	
	**Slow movements (s)**	Male	Empty vector	13	136.5 ± 27.38	<u>G:</u> $F_{(1,51)}$=0.755; p=0.389<br><u>S:</u> $F_{(1,51)}$=0.387; p=0.537	10	145.1 ± 29.21	<u>G:</u> $F_{(1,35)}$=0.448; p=0.508<br><u>S:</u> $F_{(1,35)}$=0.108; p=0.744
			A53T α-syn	15	145.7 ± 30.71		10	130.0 ± 33.18	

		Female	Empty vector	13	134.9 ± 21.68	G×S: $F_{(1,51)}$=0.147; p=0.703	10	133.4 ± 23.91	G×S: $F_{(1,35)}$=0.823; p=0.370
			A53T α-syn	14	138.3 ± 28.74		9	135.9 ± 34.02	
	**Fast movements (s)**	Male	Empty vector	13	387.5 ± 68.11	G: $F_{(1,51)}$=0.463; p=0.499<br>S: $F_{(1,51)}$=0.109; p=0.743<br>G×S: $F_{(1,51)}$=0.105; p=0.748	10	379.0 ± 57.40	G: $F_{(1,35)}$=0.309; p=0.582<br>S: $F_{(1,35)}$=0.172; p=0.681<br>G×S: $F_{(1,35)}$=0.581; p=0.451
			A53T α-syn	15	371.7 ± 60.24		10	383.1 ± 75.86	
		Female	Empty vector	13	387.3 ± 55.96		10	403.2 ± 44.05	
			A53T α-syn	14	381.8 ± 49.95		9	375.4 ± 79.31	
**Balance beam test**	**Maximum distance covered (cm)**	Male	Empty vector	13	71.03 ± 16.97	G: $F_{(1,51)}$=2.376; p=0.129<br>S: $F_{(1,51)}$=0.314; p=0.578<br>G×S: $F_{(1,51)}$=0.048; p=0.827	10	79.34 ± 1.391	G: $F_{(1,35)}$=3.214; **p=0.0816**<br>S: $F_{(1,35)}$=1.484; p=0.2312<br>G×S: $F_{(1,35)}$=0.000; p=0.9947
			A53T α-syn	15	62.66 ± 20.59		10	69.33 ± 12.15	
		Female	Empty vector	13	68.98 ± 20.97		10	72.67 ± 18.65	
			A53T α-syn	14	57.86 ± 31.34		9	62.59 ± 27.02	
	**Number of bouts (n)**	Male	Empty vector	13	8.372 ± 5.160	G: $F_{(1,51)}$=0.517; p=0.475<br>S: $F_{(1,51)}$=0.072; p=0.790<br>G×S: $F_{(1,51)}$=2.506; p=0.120	10	5.820 ± 3.568	G: $F_{(1,34)}$=3.200; **p=0.0825**<br>S: $F_{(1,34)}$=0.565; p=0.4576<br>G×S: $F_{(1,34)}$=2.338; p=0.1355
			A53T α-syn	15	11.51 ± 4.024		10	11.08 ± 4.693	
		Female	Empty vector	13	10.31 ± 5.493		10	9.340 ± 4.303	
			A53T α-syn	14	9.071 ± 5.726		8	9.663 ± 7.105	
	**Mean event bout duration (s)**	Male	Empty vector	13	1.496 ± 0.7717	G: $F_{(1,51)}$=0.717; p=0.401<br>S: $F_{(1,51)}$=0.517; p=0.475<br>G×S: $F_{(1,51)}$=3.924; **p=0.053**	10	2.180 ± 1.239	G: $F_{(1,34)}$=1.970; p=0.1696<br>S: $F_{(1,34)}$=0.566; p=0.4570<br>G×S: $F_{(1,34)}$=5.169; **p=0.0294 ($)**
			A53T α-syn	15	1.045 ± 0.3671		10	1.120 ± 0.5432	
		Female	Empty vector	13	1.045 ± 0.3012		10	1.320 ± 0.6303	
			A53T α-syn	14	1.231 ± 0.7794		8	1.600 ± 1.038	
	**Mean event bout interval duration (s)**	Male	Empty vector	13	1.730 ± 1.021	G: $F_{(1,51)}$=1.033; p=0.3143<br>S: $F_{(1,51)}$=0.803; p=0.3743	10	1.380 ± 0.9065	G: $F_{(1,34)}$=3.423; **p=0.073**<br>S: $F_{(1,34)}$=0.005; p=0.945
			A53T α-syn	15	2.731 ± 1.177		10	1.960 ± 1.182	

		Female	Empty vector	13	2.125 ± 1.204	<u>G×S:</u> $F_{(1,51)}$=3.746; **p=0.0585**	10	1.410 ± 0.5626	<u>G×S:</u> $F_{(1,34)}$=0.000; p=1.000
			A53T α-syn	14	1.791 ± 1.612		8	1.975 ± 1.112	
	**Median event bout interval duration (s)**	Male	Empty vector	13	1.130 ± 0.7796	<u>G:</u> $F_{(1,51)}$=0.766; p=0.385<br><u>S:</u> $F_{(1,51)}$=1.582; p=0.214<br><u>G×S:</u> $F_{(1,51)}$=1.154; p=0.288	10	0.760 ± 0.8222	<u>G:</u> $F_{(1,34)}$=1.087; p=0.305<br><u>S:</u> $F_{(1,34)}$=0.500; p=0.484<br><u>G×S:</u> $F_{(1,34)}$=1.447; p=0.237
			A53T α-syn	15	1.616 ± 1.221		10	0.740 ± 0.3950	
		Female	Empty vector	13	1.098 ± 0.6114		10	0.690 ± 0.2424	
			A53T α-syn	14	1.038 ± 0.9846		8	1.100 ± 0.3780	
	**Total event duration (s)**	Male	Empty vector	13	9.024 ± 2.837	<u>G:</u> $F_{(1,51)}$=0.036; p=0.850<br><u>S:</u> $F_{(1,51)}$=0.520; p=0.474<br><u>G×S:</u> $F_{(1,51)}$=0.597; p=0.443	10	7.620 ± 0.9175	<u>G:</u> $F_{(1,34)}$=1.553; p=0.221<br><u>S:</u> $F_{(1,34)}$=0.517; p=0.477<br><u>G×S:</u> $F_{(1,34)}$=0.685; p=0.414
			A53T α-syn	15	9.990 ±3.384		10	9.810 ± 3.802	
		Female	Empty vector	13	9.122 ± 3.960		10	9.210 ± 3.254	
			A53T α-syn	14	8.496 ± 4.773		8	9.638 ± 4.296	
**Pole test**	**Latency to turn (s)**	Male	Empty vector	13	4.308 ± 3.278	<u>G:</u> $F_{(1,51)}$=0.409; p=0.525<br><u>S:</u> $F_{(1,51)}$=1.790; p=0.187<br><u>G×S:</u> $F_{(1,51)}$=0.256; p=0.615	10	3.330 ± 3.162	<u>G:</u> $F_{(1,35)}$=0.640; p=0.429<br><u>S:</u> $F_{(1,35)}$=1.127; p=0.296<br><u>G×S:</u> $F_{(1,35)}$=0.656; p=0.423
			A53T α-syn	15	1.955 ± 0.8813		10	3.510 ± 1.821	
		Female	Empty vector	13	6.001 ± 9.657		10	4.490 ± 6.050	
			A53T α-syn	14	5.786 ± 11.94		9	11.60 ± 26.86	
	**Descent time (s)**	Male	Empty vector	13	6.397 ± 1.407	<u>G:</u> $F_{(1,51)}$=0.966; p=0.330<br><u>S:</u> $F_{(1,51)}$=0.875; p=0.354<br><u>G×S:</u> $F_{(1,51)}$=0.678; p=0.414	10	5.900 ± 1.267	<u>G:</u> $F_{(1,35)}$=1.235; p=0.274<br><u>S:</u> $F_{(1,35)}$=1.578; p=0.217<br><u>G×S:</u> $F_{(1,35)}$=1.055; p=0.311
			A53T α-syn	15	6.802 ± 5.533		10	6.500 ± 2.109	
		Female	Empty vector	13	6.538 ± 5.841		10	6.960 ± 2.069	
			A53T α-syn	14	10.45 ± 13.35		9	16.12 ± 26.93	
	**Total time on pole (s)**	Male	Empty vector	13	10.70 ± 3.809	<u>G:</u> $F_{(1,51)}$=0.063; p=0.802<br><u>S:</u> $F_{(1,51)}$=2.644; p=0.110	10	9.250 ± 3.835	<u>G:</u> $F_{(1,35)}$=0.927; p=0.342<br><u>S:</u> $F_{(1,35)}$=1.366; p=0.250
			A53T α-syn	15	8.756 ± 5.831		10	10.01 ± 2.609	

		Female	Empty vector	13	12.54 ± 11.23	G×S: $F_{(1,51)}$=0.908; p=0.345	10	11.46 ± 7.350	G×S: $F_{(1,35)}$=0.858; p=0.361
			A53T α-syn	14	16.24 ± 17.48		9	27.72 ± 53.71	

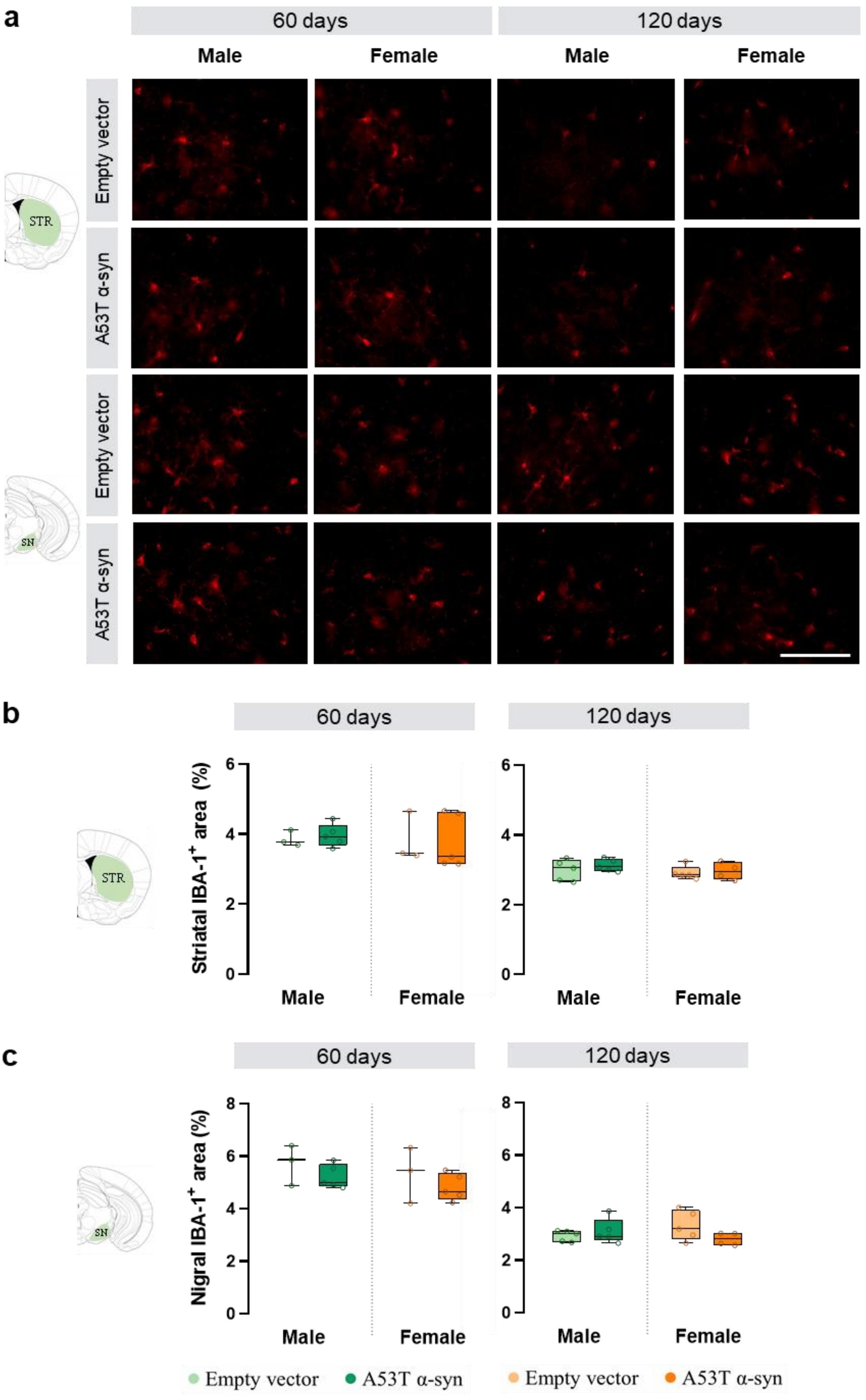
a
60 days
120 days
Male
Female
Male
Female
Empty vector
A53T α-syn
Empty vector
A53T α-syn
STR
SN
b
60 days
120 days
Striatal IBA-1+ area (%)
Male
Female
Male
Female
c
60 days
120 days
Nigral IBA-1+ area (%)
Male
Female
Male
Female
Empty vector
A53T α-syn
Empty vector
A53T α-syn

Fig. S8 Absence of microglial activation in the nigrostriatal pathway. (a) Representative images showing ionized calcium-binding adapter molecule 1 (IBA-1) immunofluorescence in the striatum and *substantia nigra* of empty vector and α-synuclein (α-syn) animals at 60 and 120 days post-surgery. Scale bar: 80 µm. **(b)** Quantification of IBA-1 expression revealed no significant changes in microglial activation across groups or time points. 60 days: male empty vector, n = 3; male α-syn, n = 5; female empty vector, n = 3; female α-syn, n = 5; 120 days: male empty vector, n = 5; male α-syn, n = 5; female empty vector, n = 5; female α-syn, n = 4
